# Integrative multi-omics analysis prioritizes compartment-specific candidate targets of gut microbiota metabolites in diabetic kidney disease

**DOI:** 10.3389/fimmu.2026.1925910

**Published:** 2026-08-31

**Authors:** Fengwei Xie, Xiongwei He, Wenjie Chen, Jiahao Li, Sihan Lin, Qihang Bao, Weiqing Li, Junpeng Cheng, Yuan Ma, Ziyu Wu, Haohuan Li

**Affiliations:** 1 Department of Orthopedics, Renmin Hospital of Wuhan University, Wuhan, China; 2 Department of Nephrology, Renmin Hospital of Wuhan University, Wuhan, China; 3 Department of Nephrology, Huzhou Central Hospital, Second School of Clinical Medicine of Zhejiang Chinese Medical University, Huzhou, China

**Keywords:** diabetic kidney disease, gut microbiota, gutMGene database, multi-omics analysis, therapeutic targets

## Abstract

**Objective:**

The gut-kidney axis has emerged as a critical area of investigation. However, the compartment-specific regulatory mechanisms of gut microbiota metabolites within the glomerular and tubulointerstitial regions of diabetic kidney disease (DKD) remain incompletely understood.

**Methods:**

The gutMGene, SEA, STP, GEO, Nephroseq v5, and KIT databases were interrogated. Multi-omics and experimental approaches were applied, involving differential expression analysis, Weighted Gene Co-expression Network Analysis (WGCNA), machine learning, Mendelian randomization (MR), Gene Set Enrichment Analysis (GSEA), immune infiltration, gene set variation analysis (GSVA), clinical correlation, single-cell profiling, molecular docking, molecular dynamics simulation, CCK-8 assay, and RT-qPCR. The “Microbiota-Substrate-Metabolite-Target” (M-S-M-T) network was constructed for each compartment.

**Results:**

Machine learning identified six glomerular (*IGFBP6*, *PLA2G4A*, *CTSK*, *HTR2B*, *PDGFRA*, *MMP7*) and five tubulointerstitial (*CA2*, *HSD11B2*, *NQO2*, *MMP7*, *CYP24A1*) core genes. Mendelian randomization, single-cell profiling, and clinical cohorts provided supportive genetic and clinical evidence linking these genes to the estimated glomerular filtration rate (eGFR) and to DKD. Immune infiltration analysis and GSVA indicated a higher estimated proportion of M2 macrophages and a shift of the estimated mast cell composition from activated to resting states in both compartments, which correlated with core gene expression. Via the “M-S-M-T” network, drug-likeness and toxicity screening, caffeic acid and naringenin chalcone were prioritized as shared candidate metabolites. Molecular docking and molecular dynamics simulations suggested favorable in silico binding to HTR2B and MMP7. Both metabolites attenuated high glucose (HG)-induced core gene dysregulation in HK-2 cells and podocytes.

**Conclusion:**

This study proposed a compartment-specific “M-S-M-T” regulatory network in the DKD glomerulus and tubulointerstitium. The shared metabolites caffeic acid and naringenin chalcone, which targeted HTR2B and MMP7, produced a preliminary protective transcriptional response *in vitro*. These findings are hypothesis-generating and require validation in real-world microbiome, metabolomic and *in vivo* studies before any therapeutic inference can be drawn.

## Highlights

- Compartment-specific core genes were identified in the DKD glomerulus (6 genes) and tubulointerstitium (5 genes) using multi-omics approaches.
- The dysregulation of core genes correlated with computationally estimated immune alterations, particularly a higher M2 macrophage fraction and a mast cell phenotypic shift.
- Compartment-specific “Microbiota-Substrate-Metabolite-Target” (M-S-M-T) regulatory networks were constructed for the glomerular and tubulointerstitial regions in DKD.
- Integrating molecular docking, molecular dynamics simulations, and cell experiments, caffeic acid and naringenin chalcone were prioritized as shared candidate metabolites that merit further experimental validation in DKD.

## 1 Introduction

Diabetic kidney disease (DKD) is the primary etiology of end-stage renal disease (ESRD) (1). While recent clinical trials utilizing Sodium-Glucose Cotransporter 2 (SGLT2) inhibitors (2), Glucagon-Like Peptide-1 Receptor Agonists (GLP-1 RAs) (3), and non-steroidal Mineralocorticoid Receptor Antagonists (ns-MRAs) (4) have demonstrated cardiorenal protection independent of glucose-lowering, the precise mechanisms remain partially elucidated. This suggests that inter-organ crosstalk constitutes a pivotal therapeutic axis for mitigating DKD progression.

The gut-kidney axis has garnered extensive research interest as a critical mechanism of inter-organ communication relevant to DKD pathogenesis (5). Clinical investigations consistently reveal significant gut microbial dysbiosis in the fecal samples of patients with DKD, with these alterations demonstrating strong correlations with clinical indicators of lipid metabolism, glucose homeostasis, and renal function (6). This ecological imbalance is not confined to DKD, having been systematically supported in both diabetic and non-diabetic chronic kidney disease cohorts (7). Functionally, aberrations in microbial metabolites drive disease; for example, diminished short-chain fatty acids (SCFAs) correlate with ESRD risk (8). Indole-3-propionic acid (IPA) confers mitochondrial protection via SIRT1 (9). Furthermore, therapeutic modulation of the gut microbiota has proven efficacy in DKD models (10, 11).

**Figure 1:**
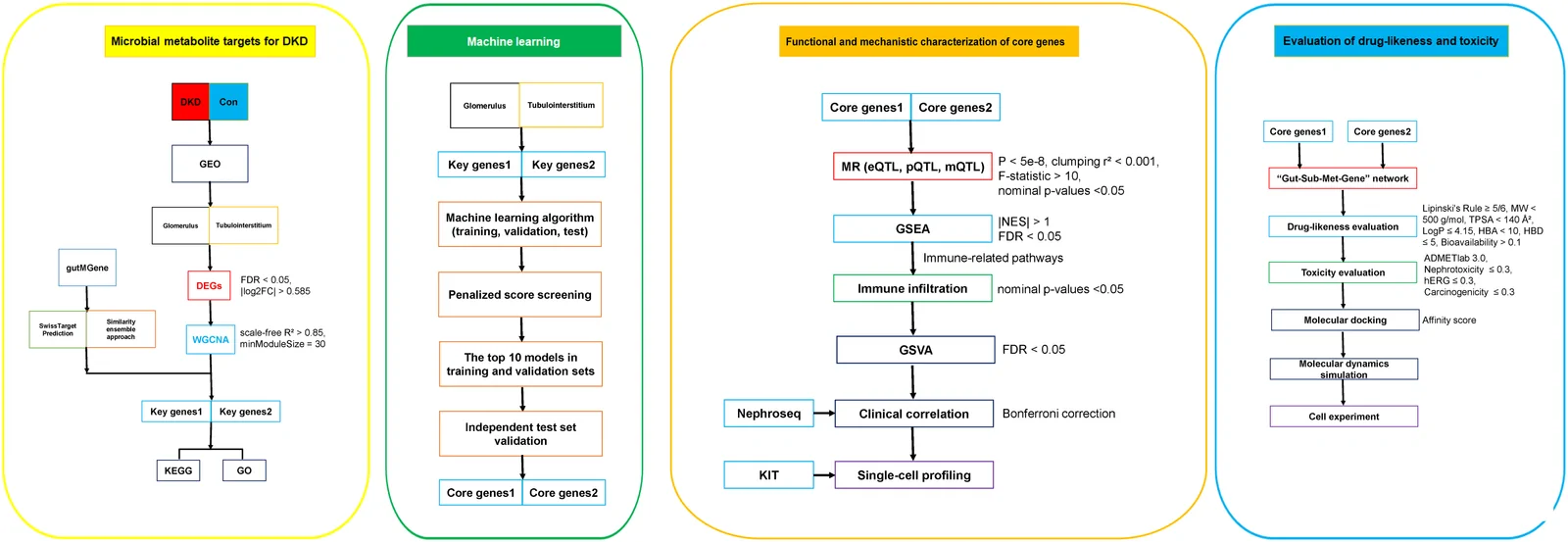
Flow-chart of this paper.

Although computational methodologies have been used to explore the gut microbiota-metabolite-target network in DKD (12), the kidney’s cellular heterogeneity poses a challenge. Target identification relying on general databases, such as GeneCards (13), cannot adequately resolve whether key pathological events originate in the glomerular or tubulointerstitial compartments. To address these limitations, our study utilized compartment-specific datasets and advanced bioinformatics to construct a resolved regulatory network. The workflow with the inclusion thresholds was summarized in **Figure 1**. It should be stated at the outset that this is a computational, hypothesis-generating study whose mechanistic center was the compartment-resolved “Microbiota-Substrate-Metabolite-Target” (M-S-M-T) relationship; the immune and therapeutic observations were supportive and correlative rather than mechanistically established.

## 2 Materials and methods

### 2.1 Data source

Detailed database information is presented in **Supplementary Table 1**.

### 2.2 Acquisition of DKD-related targets

Ten transcriptomic datasets were retrieved from the NCBI Gene Expression Omnibus (GEO) database (https://www.ncbi.nlm.nih.gov/gds) (accessed 13 September 2025). Glomerular datasets were divided into training (GSE104948, GSE96804, GSE47183), validation (GSE99339), and independent test cohorts (GSE30528, GSE142025). Tubulointerstitial datasets were similarly partitioned: training (GSE104954, GSE30529), validation (GSE99325), and test (GSE47184).

### 2.3 Identification of gut microbiota metabolite targets

Gut microbiota metabolites were retrieved from the gutMGene database (14) (accessed 4 September 2025) (filtered by *Homo sapiens* and molecular weight), yielding 248 compounds. Targets were predicted using SwissTargetPrediction (STP) (15) (accessed 4 September 2025) and Similarity Ensemble Approach (SEA) (16) (accessed 4 September 2025), with intersections visualized via the ggvenn (v0.1.10) (17).

### 2.4 Differentially expressed genes analysis

DEGs were calculated using the limma package (18) (FDR < 0.05, |log2FC| > 0.585).

### 2.5 Weighted gene co-expression network analysis

WGCNA (v1.73) (19) was performed to construct a scale-free network (R² > 0.85). Modules were mapped via dynamic tree cutting (minModuleSize = 30) and correlated with clinical phenotypes.

### 2.6 Protein–protein interaction network

PPI networks were constructed using the STRING database (20) (accessed 11 September 2025).

### 2.7 GO and KEGG enrichment analysis

Kyoto Encyclopedia of Genes and Genomes (KEGG) (21) and Gene Ontology (GO) (22) enrichment analyses were conducted with a standard significance threshold (p-value < 0.05, q-value < 0.05).

### 2.8 Machine learning and SHAP analysis

Before modelling, datasets were merged, batch-corrected with ComBat, and scaling parameters were fitted on the training set only to prevent data leakage. We constructed 137 models using twelve machine learning algorithms (Lasso, XGBoost, Stepglm, SVM, LDA, GBM, glmBoost, Enet, RF, plsRglm, Ridge, and NaiveBayes), performing algorithm-specific cross-validation and model-specific hyperparameter tuning. For composite models, feature-ranking steps retained default parameters to avoid overfitting and ensure computational feasibility. Class balance was evaluated in both compartments (glomerular: 48.5% cases; tubulointerstitial: 41.5% cases), with no resampling or class weighting applied. Model efficacy was measured by the Area Under the Curve (AUC).

To penalize for overfitting, we utilized a specific score for model selection:

Penalized Score = Average Validation AUC − 2 × (Train AUC − Average Validation AUC).

The optimal model underwent SHAP analysis to quantify feature importance.

Feature stability was confirmed by bootstrap resampling (100 iterations), with genes selected in >80% of resamples considered stable.

### 2.9 Single-gene ROC curve evaluation

The pROC package (v1.19.0.1) was employed to calculate AUC for individual genes across each respective dataset.

### 2.10 MR analysis

Two-sample MR was performed using the TwoSampleMR package (v0.6.22). Cis-eQTL, cis-pQTL, and cis-mQTL instruments were retrieved from the eQTLGen consortium, the deCODE genetics and UK Biobank Pharma Proteomics Project studies, and the Genetics of DNA Methylation Consortium, respectively. Instrumental variables were filtered by significance (P < 5e-8), independence (clumping r^2^ < 0.001, window size = 10000 kb), and strength (F-statistic > 10). Outcomes were sourced from OpenGWAS. Sensitivity analyses comprised Cochran’s Q test for heterogeneity, the MR-Egger intercept test for horizontal pleiotropy, leave-one-out analysis and funnel plots.

### 2.11 Single-gene gene set enrichment analysis

According to the medium expression of each gene, samples were divided into two groups. Via DEGs analysis and the C2 (c2.cp.kegg_legacy) collection of the Molecular Signatures Database (MSigDB), normalized enrichment score was calculated for each pathway.

### 2.12 Immune cell infiltration analysis

Immune cell infiltration was quantified in all datasets using the CIBERSORT algorithm (23) (accessed 20 September 2025). Results were visualized using heatmaps (immune cell correlations), box plots (comparative analyses), and lollipop plots (gene-immune cell associations).

### 2.13 Gene set variation analysis

Gene sets were extracted from the C5 GO: BP collection of MSigDB. Gene sets with at least 10 overlapping genes in the expression matrix were retained. 7 macrophage-related and 3 mast cell-related pathways were selected, and GSVA (ssGSEA method) was applied to calculate pathway enrichment scores for each sample. Heatmaps and Lollipop plots illustrated the association between core gene expression and enrichment scores of selected pathways.

### 2.14 Online database analysis

Data from Nephroseq v5 (24, 25) (accessed 20 September 2025) were used to correlate core gene expression with estimated glomerular filtration rate (eGFR) in DKD glomerular and tubulointerstitial compartments. The Kidney Interactive Transcriptomics platform (26–28) (accessed 20 September 2025) was used to analyze cell-type-specific differential expression.

### 2.15 Analysis of drug-likeness and toxicity

Candidate metabolite physicochemical properties were derived from the SwissADME platform (29) based on their respective SMILES notations. For drug-likeness, we applied Lipinski’s Rule, retaining compounds that met at least five of the following criteria: molecular weight <500 g/mol, topological polar surface area (TPSA) <140 Å², lipid–water partition coefficient ≤4.15, number of hydrogen bond acceptors <10, and number of hydrogen bond donors ≤5. A bioavailability score >0.1 was also required (30). The ADMETlab 3.0 was subsequently used to discard compounds with predicted nephrotoxicity, arrhythmia risk or carcinogenicity (probability threshold 0.3) (31).

### 2.16 Molecular docking

The 3D structures of the target proteins were retrieved from the RCSB Protein Data Bank (PDB) and the AlphaFold Protein Structure Database. Specifically, the PDB entries utilized were 1BCI (PLA2G4A), 7QBM (CTSK), 6DRX (HTR2B), 8PQH (PDGFRA), 1MMQ (MMP7), 1CA3 (CA2), 4QOD (NQO2), and 2JM2 (IGFBP6). For proteins lacking experimental crystal structures, predicted models were employed for HSD11B2 (AF_AFP80365F1) and CYP24A1 (AF_AFQ07973F1). The 3D structures of the ligands were obtained from the PubChem database (32). During the docking preparation, ion pair effects were specifically excluded for CA2 and CTSK interactions. Ligand formats and results were processed using Open Babel (v3.1.1). The protein-ligand interactions were analyzed using the Protein-Ligand Interaction Profiler (PLIP) and visualized using the open-source version of PyMOL (3.2.0a0 master). Binding affinities were summarized and presented as a heatmap.

### 2.17 Molecular dynamics simulation

Molecular dynamics simulations were executed using GROMACS 2025.2 and the CHARMM36 force field. The protein-ligand complex was placed in a cubic box, solvated with the TIP3P water model, and neutralized with 150 mM NaCl. The protocol involved energy minimization, 100 ps NVT/NPT equilibrations, and a 100 ns production run. Trajectory analysis included backbone Cα RMSD, Rg, RMSF, SASA, and hydrogen bond counts. The Molecular Mechanics/Generalized Born Surface Area (MM/GBSA) method was used to calculate the binding free energy (ΔG_bind_) and perform per-residue decomposition to map binding hotspots. Finally, Molecular Electrostatic Potential (ESP) and Free Energy Landscapes (FELs) were used to analyze the ligand’s charge distribution and conformational stability, respectively.

### 2.18 Cell line and reagents

Human renal proximal tubular epithelial cells (HK-2 cells) were obtained from the American Type Culture Collection (Rockville, MD, USA) and maintained in a 1:1 mixture of Dulbecco’s Modified Eagle Medium and Ham’s F-12 nutrient medium containing 5% fetal bovine serum. Conditionally immortalized human podocytes (HPC) were kindly provided by Dr. Moin A. Saleem (Academic Renal Unit, Southmead Hospital, Bristol, UK) and cultured in RPMI 1640 medium (SH30809.01, HyClone, USA) supplemented with 10% fetal bovine serum (FBS, 10100; Gibco, USA), penicillin G (100 U/ml), streptomycin (100 μg/ml) (15140122; Life, USA), and 1× insulin-transferrin-selenium (ITS, 41400045; Life, USA) at 33 °C for proliferation. When the density of HPC reached 70–80%, these cells were transferred to 37 °C with an ITS-free medium for differentiation (33). All cells were routinely checked for mycoplasma contamination while being cultivated at 37 °C and 5.0% CO2. Naringenin chalcone and caffeic acid were purchased from MedChemExpress (Shanghai, China).

### 2.19 RNA extraction and quantitative real-time PCR analysis

Total RNA was isolated using TRIzol reagent (Invitrogen, USA). All primers (**Table 1**) were designed based on NCBI reference sequences and synthesized by Shenzhen Huada Gene Science and Technology Service (Shanghai, China). mRNA expression levels were normalized to GAPDH. Relative mRNA expression was quantified using the 2^−ΔΔCt^ method.

**Table 1:** Primer pairs of target genes used for RT-qPCR.

Cell type	Gene	Forward primer (5′→3′)	Reverse primer (5′→3′)
HPC	IGFBP6	ACATCTGGACTCAGTGCTGC	TGGTCACAATTGGGCACGTA
HPC	CTSK	GGGGGACATGACCAGTGAAG	CAGAGTCTGGGGCTCTACCT
HPC	PLA2G4A	TCTGCAAGGCCAAGTGACTC	TCAGCCCTTCCCGATCAAAC
HPC	HTR2B	TCCGGAATCCAATGGCAGAG	ACTTCGGAGCCTCATTGGAC
HPC	PDGFRA	ACTCCCGAGACAGGAGTACC	CTCGGTTCTCAGCTCCAAGG
HPC	MMP7	CAGGAAACACGCTGGCTCAT	CCCTAGACTGCTACCATCCG
HK-2	MMP7	CAGGAAACACGCTGGCTCAT	CCCTAGACTGCTACCATCCG
HK-2	HSD11B2	GGCAAGGACTACATCGAGCA	AGGCCTTCAGGCAGGTAGTA
HK-2	CYP24A1	GAAGGCCTATCGCGACTACC	GCTTCATCACTTCCCCTGGT
HK-2	CA2	TCCTTGGATTACTGGACCTACC	CGGAATTTCAACACCTGCTC
HK-2	NQO2	TGCCCCTCAGATCAGCTTTG	TTATTGCCCGAAGTGCCAGT

### 2.20 Cell viability assay and drug concentration screening

To determine the optimal working concentrations of naringenin chalcone and caffeic acid, HPC and HK-2 cells were seeded into 96-well plates at a density of 5,000 cells per well and cultured overnight for attachment. Subsequently, the cells were treated with a series of concentrations of naringenin chalcone (0, 5, 10, 30, 50, 100, and 200 μM) or caffeic acid (0, 5, 10, 30, 50, and 100 μM) for 24 h. Cell viability was assessed using the Cell Counting Kit-8 (CCK-8, Beyotime Biotechnology, Shanghai, China). Briefly, 10 μL of CCK-8 solution was added to each well, followed by incubation at 37 °C for 2 h. The absorbance values were measured at 450 nm using a microplate reader (Tecan, Switzerland). The relative cell viability was calculated as a percentage of the control group, and the concentrations showing no significant cytotoxicity were selected for subsequent experiments. To simulate the diabetic microenvironment *in vitro*, HPC and HK-2 cells were exposed to 25 mM high glucose for 24 hours to induce cellular injury. Cells cultured in 5 mM glucose served as the normal control group. To exclude the effect of hyperosmotic stress, an osmotic control group was included using 5 mM glucose supplemented with 20 mM mannitol. For drug treatment, cells were co-incubated with high glucose and the indicated concentrations of naringenin chalcone or caffeic acid or inhibitors for 24 hours.

### 2.21 Statistical analysis

Data were analyzed using GraphPad Prism 11 software and R 4.5.1. Values are presented as mean ± SD. Statistical comparisons of groups were performed using 1-way analysis of variance, and multiple comparisons of groups were performed using the least-significant difference test. P < 0.05 was considered statistically significant. For high-dimensional analyses, FDR (Benjamini–Hochberg) was applied to DEGs, GSEA, and GSVA. Correlations between core genes and eGFR were Bonferroni-corrected across 11 tests. Immune infiltration and MR results are reported at nominal significance (exploratory). Normality was assessed by Shapiro–Wilk and homogeneity by Levene’s test, with non-parametric or Welch’s ANOVA used as appropriate. For pre-specified pairwise comparisons (≤4 groups), the LSD test was used.

### 2.22 Western blot

HK-2 cells or podocytes were washed three times with phosphate-buffered saline and lysed in WB/IP lysis buffer (Solarbio, Beijing, China) supplemented with 1% (v/v) protease inhibitor cocktail and 1% (v/v) phosphatase inhibitor cocktail for 30 min on ice. After centrifugation, the supernatants were collected, and protein concentrations were determined using a bicinchoninic acid (BCA) assay kit (Elabscience, #E-BC-K318-M) at 562 nm. Equal amounts of protein were adjusted with lysis buffer, mixed with SDS-PAGE loading buffer, and boiled at 100 °C for 10 min. Proteins were then separated by 12.5% SDS-PAGE and transferred onto polyvinylidene difluoride membranes (Millipore, USA). The membranes were blocked with 5% non-fat milk for 2 h at room temperature and incubated overnight at 4 °C with primary antibodies against HTR2B (Abcam, ab194333, 1:1000), MMP7 (Abcam, ab207299, 1:1000), Bcl-2 (Proteintech, 68103-1-Ig, 1:1000), and β-Actin (Proteintech, 66009-1-Ig, 1:5000). After three washes with Tris-buffered saline containing 0.1% Tween-20, the membranes were incubated with horseradish peroxidase-conjugated goat anti-rabbit or anti-mouse IgG (1:10000) for 1 h at room temperature. Protein bands were visualized using an enhanced chemiluminescence detection system (R-tracker ECL, Yeasen, Shanghai, China). Band intensities were quantified using ImageJ software (NIH, USA) and normalized to β-Actin as an internal control.

## 3 Results

### 3.1 Stepwise filtering of glomerular and tubulointerstitial DKD targets linked to gut microbiota metabolites

**Figure 2:**
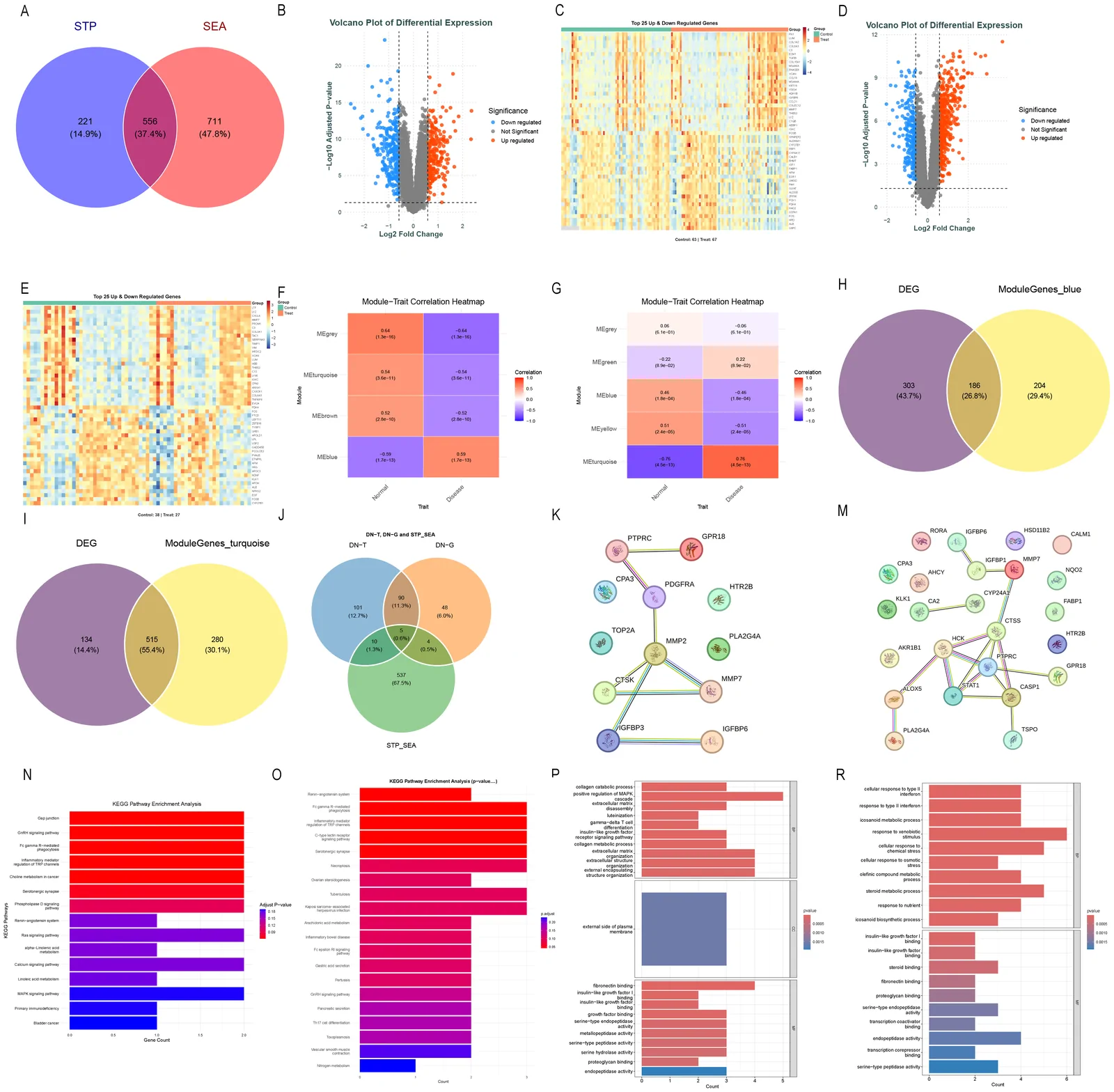
Stepwise filtering of glomerular and tubulointerstitial DKD targets linked to gut microbiota metabolites. **(A)** Venn diagram illustrating the predicted targets of gut microbiota metabolites, identified using the STP and SEA databases. **(B)** Volcano plot for the glomerular and **(D)** tubulointerstitial cohorts showing differentially expressed genes (DEGs); red dots represent upregulated genes, and blue dots represent downregulated genes. **(C)** Heatmap for the glomerular and **(E)** tubulointerstitial cohorts illustrating the expression profiles of DEGs grouped by sample status (Group); red indicates upregulation, and blue indicates downregulation. **(F)** Module-trait correlation heatmap for the glomerular and **(G)** tubulointerstitial cohorts, showing the correlation between gene modules and clinical traits. **(H)** Venn diagram for the glomerular and **(I)** tubulointerstitial cohorts, showing the overlapping genes between DEGs (purple) and the most relevant gene module (yellow). **(J)** Venn diagram illustrating the intersection of core genes from the glomerular cohort (orange), the tubulointerstitial cohort (blue), and the targets of gut microbiota metabolites (green). **(K)** PPI network constructed via STRING for the glomerular and **(M)** tubulointerstitial core targets, showing protein–protein interactions among the filtered gene sets. **(N)** KEGG pathway enrichment analysis and **(P)** GO enrichment analysis for the overlapping genes in the glomerular cohort. **(O)** KEGG pathway enrichment analysis and **(R)** GO enrichment analysis for the overlapping genes in the tubulointerstitial cohort. For **(N–P, R)**, the x-axis showing the number of genes, and the color gradient representing the adjusted p-value. These analyses progressively narrow the initial gene pools to gut-microbiota-related candidate targets specific to each renal compartment.

We initiated the analysis by screening 248 gut microbiota metabolites from the gutMGene database. The SEA and STP databases yielded 1267 and 777 targets, respectively, resulting in 556 shared target genes after intersection (**Figure 2A**).

As previously described, training sets for the glomerular and tubulointerstitial compartments in DKD were established after batch effect removal, correction, and merging. Differential expression analysis was performed on each dataset. In the glomerular dataset, a volcano plot highlighted 265 downregulated and 224 upregulated genes (**Figure 2B**), while the tubulointerstitial dataset contained 248 downregulated and 401 upregulated genes (**Figure 2D**). Heatmaps depicted the expression profiles of the top 25 upregulated and downregulated genes in both datasets (**Figures 2C, E**).

Utilizing the WGCNA package, we determined soft-thresholding powers of 9 (glomerular dataset) and 6 (tubulointerstitial dataset) and dynamically partitioned gene modules based on co-expression relationships (**Supplementary Figures 1A, C**). A Topological Overlap Matrix (TOM) was constructed to identify co-expression modules (**Supplementary Figures 1B, D**). Module-trait relationship heatmaps delineated 4 and 5 modules significantly associated with DKD in the glomerular and tubulointerstitial datasets, respectively (**Figures 2F, G**). Notably, the “MEblue” module in the glomerular dataset showed the highest correlation with the disease trait (*r* = 0.59, *p* = 1.7e-13), while the “MEturquoise” module displayed the strongest correlation in the tubulointerstitial dataset (*r* = 0.76, *p* = 4.5e-13).

Intersecting these significant module genes with the previously identified differentially expressed genes (DEGs), we narrowed the lists to 186 genes for the glomerulus (**Figure 2H**) and 515 genes for the tubulointerstitium (**Figure 2I**). Further intersected with the 556 shared metabolite targets, it yielded 9 key genes for the glomerular compartment and 15 for the tubulointerstitial compartment (**Figure 2J**). PPI networks based on the STRING database illustrated the interactions among these final gene sets (**Figures 2K–M**). Subsequent KEGG (**Figures 2N, O**) and GO (**Figures 2P–R**) enrichment analyses were performed. KEGG analysis uncovered several pathways significantly enriched in both compartments, including the GnRH signaling pathway, Fc gamma R-mediated phagocytosis, Inflammatory mediator regulation of TRP channels, Serotonergic synapse, and the Renin-angiotensin system. This implies shared pathological mechanisms between the glomerulus and tubulointerstitium in DKD. In the glomerular dataset, the most significant GO terms were “collagen catabolic process” (BP), “external side of plasma membrane” (CC), and “fibronectin binding” (MF). In the tubulointerstitial dataset, the most significant terms were “cellular response to type II interferon” (BP) and “insulin-like growth factor I binding” (MF).

### 3.2 Machine learning of core genes with cross-cohort generalizability

**Figure 3:**
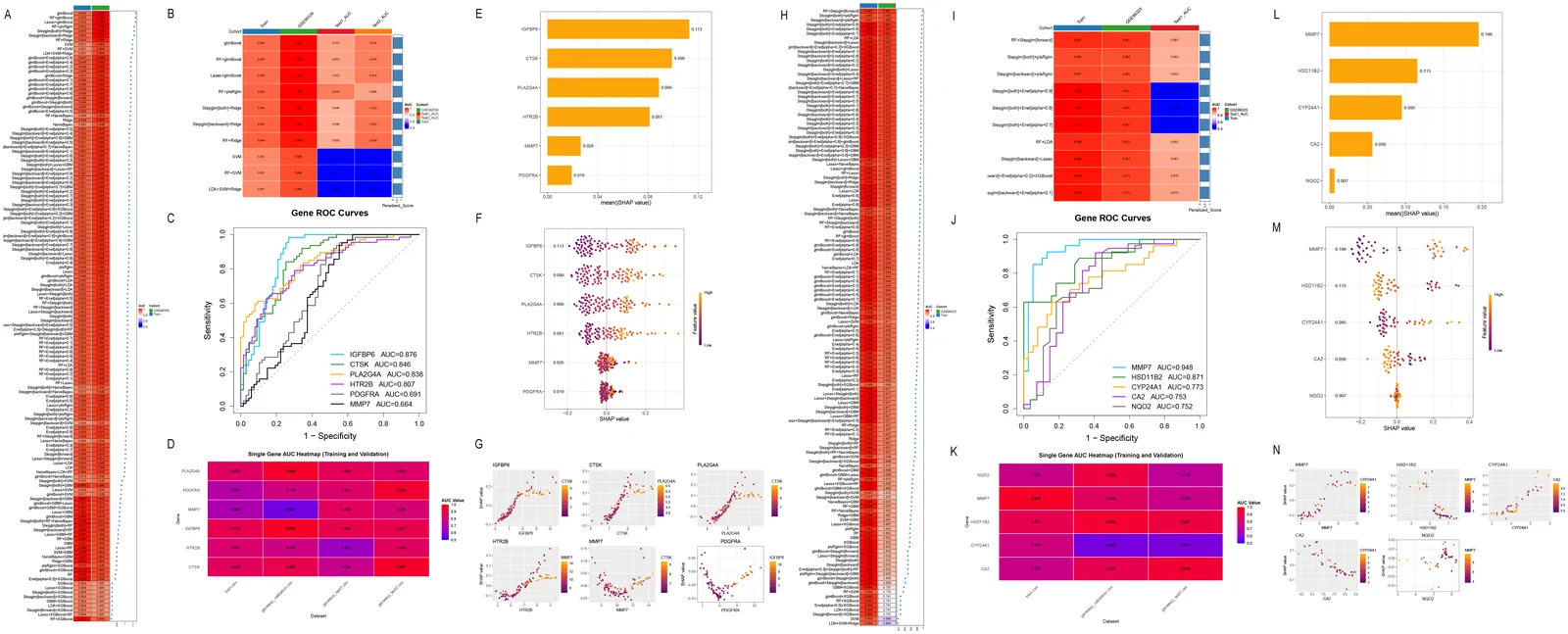
Machine learning of core genes with cross-cohort generalizability. **(A)** Heatmap for the glomerular and **(H)** tubulointerstitial cohorts, illustrating the Area Under the Curve (AUC) values of 137 machine learning models on the training and validation sets, ordered by the penalized score. **(B)** AUC performance of the top ten models across the training, validation, and independent test sets for the glomerular and **(I)** tubulointerstitial cohorts. **(C)** Single-gene Receiver Operating Characteristic (ROC) curves for the core genes on the training set in the glomerular and **(J)** tubulointerstitial cohorts. **(D)** Heatmap of single-gene AUC performance for the core genes across the training, validation, and independent test sets in the glomerular and **(K)** tubulointerstitial cohorts. **(E)** SHAP Summary Plot for the glomerular and **(L)** tubulointerstitial cohorts, demonstrating the mean absolute contribution |SHAP value| of each core gene to the model output. **(F)** Beeswarm Plot for the glomerular and **(M)** tubulointerstitial cohorts, showing the relationship between the SHAP value and the feature value (gene expression level) for each core gene. **(G)** SHAP Dependence Plots for the glomerular cohort, showing scatter plots of core gene expression levels versus SHAP values. The color gradient represents the expression of PLA2G4A, MMP7, IGFBP6, and CTSK. The co-expression pattern of IGFBP6 and CTSK suggests a potential synergistic contribution to DKD prediction. **(N)** SHAP Dependence Plots for the tubulointerstitial cohort, showing scatter plots of core gene expression levels versus SHAP values. The color gradient represents the expression of CYP24A1, MMP7, and CA2. The multi-model approach prioritized genes with favorable diagnostic performance in the training set, which were then evaluated across independent cohorts for generalizability.

We first applied machine learning to the glomerular dataset to screen for core genes (**Figure 3A**). The top ten models were selected based on penalized scores. These models were then evaluated on two independent test sets to assess their generalization ability (**Figure 3B**). Based on this, we selected the top three models, which demonstrated superior and robust generalization in the test sets. Notably, all three models highlighted the same set of core genes: *IGFBP6*, *CTSK*, *PLA2G4A*, *HTR2B*, *PDGFRA*, and *MMP7*. Box plots illustrated the expression patterns of these genes in the glomeruli of DKD versus control samples across all datasets. The expression changes for each core gene in the validation and test sets were consistent with those in the training set, although a few cases were not statistically significant (**Supplementary Figures 2A–D**). These six core genes were markedly upregulated. Using the pROC package, single-gene ROC curves were generated for the training set (**Figure 3C**). We further calculated the single-gene AUC performance in the validation and test sets, summarizing these in an AUC heatmap (**Figure 3D**). Among these, *PLA2G4A* and *IGFBP6* indicated excellent and robust diagnostic utility (AUC > 0.82). The SHAP summary plot showed *IGFBP6* and *CTSK* to be the primary contributors to the model’s output (**Figure 3E**). Beeswarm plots showed that *IGFBP6*, *CTSK*, *PLA2G4A*, and *HTR2B* exerted a positive impact on the disease prediction (**Figure 3F**), while *MMP7* and *PDGFRA* had a bidirectional effect. Dependence plots further supported these findings and unveiled a synergistic effect on the prediction from the co-high expression of *IGFBP6* and *CTSK* (**Figure 3G**). SHAP analyses of the validation and test sets reinforced the critical roles of *IGFBP6* and *CTSK* in the model (**Supplementary Figures 3A–I**).

Similarly, this process was replicated for the tubulointerstitial dataset. Machine learning isolated the top ten models (**Figure 3H**), which were then assessed for generalization ability on test sets (**Figure 3I**). The final four models performed optimally, pinpointing the same core genes: *MMP7*, *HSD11B2*, *CYP24A1*, *CA2*, and *NQO2*. Box plots illustrated the expression patterns of these genes in the tubulointerstitium of DKD versus control samples. Expression changes in the validation and test sets were consistent with the training set, though some were not significant (**Supplementary Figures 2E–G**). As described previously, single-gene ROC curves for the training set (**Figure 3J**) and an AUC heatmap for all datasets (**Figure 3K**) were generated. *HSD11B2* showed an excellent diagnostic value (AUC > 0.87).

The SHAP summary plot indicated that *MMP7* was the primary contributor to the model, while *NQO2* had the smallest relative contribution (**Figure 3L**). Beeswarm plots revealed that *MMP7* and *CYP24A1* positively influenced the disease prediction, whereas *HSD11B2* and *CA2* had a negative impact (**Figure 3M**). Dependence plots were consistent with these conclusions and supported a synergistic effect between *MMP7* and *CYP24A1* (**Figure 3N**). Finally, SHAP analyses of the validation and test sets indicated the key role of *MMP7* in the model (**Supplementary Figures 3J–O**).

### 3.3 Supportive genetic evidence for core genes from MR

We performed MR with five DKD-related renal traits through eQTL, pQTL, and mQTL genetic instruments (**Supplementary Figure 4**, **Supplementary Table 2**). Regarding risk factors, genetically predicted higher expression of *NQO2* was significantly associated with reduced eGFR in eQTL analysis (OR = 0.999, *p* = 0.032), supported by its mQTL against renal failure (OR = 0.953, 95%CI: 0.918–0.990, *p* = 0.014). Elevated expression of *CTSK* (eQTL) and higher plasma levels of *PLA2G4A* (pQTL) were tied to reduced eGFR (*CTSK*: OR = 0.998, p < 0.001; *PLA2G4A*: OR = 0.992, p = 0.046). Their corresponding mQTLs revealed supportive genetic evidence with reduced eGFR (*CTSK*: OR = 0.995, p = 0.014; *PLA2G4A*: OR = 0.987, p < 0.001), suggesting that SNP-related CpG sites may be located in transcriptionally active gene-body regions.

Conversely, *IGFBP6* (eQTL) was associated with chronic kidney disease (OR = 0.700, p = 0.028) and renal failure (OR = 0.739, p = 0.022), with epigenetic evidence (mQTL) indicating the same effect on reduced eGFR and DKD. This may be attributed to single-SNP bias. The shared target *MMP7*(pQTL) showed supportive genetic association with preserved eGFR in both UKBPPP and deCODE.

For *HTR2B*, mQTL analysis linked to reduced eGFR (OR = 0.997, *p* < 0.001), accompanied by increased risk of DKD (OR = 1.153, *p* = 0.038). In contrast, *HSD11B2* and *CYP24A1* exhibited significant protective causal effects: *HSD11B2* methylation preserved eGFR (OR = 1.038, *p* < 0.001); the higher expression of *CYP24A1* (eQTL) was protective against DKD (OR = 0.607, *p* = 0.013). Interestingly, *PDGFRA* (pQTL) was positively associated with renal failure (OR = 1.171, *P* = 0.046) and negatively associated with microalbuminuria (OR = 0.823, *P* = 0.023), suggesting stage-specific effects. Sensitivity analyses for instruments with multiple SNPs demonstrated the robustness of these estimates. Collectively, this eQTL, pQTL, and mQTL evidence robustly suggests that the core gene signature may drive key pathological processes in DKD.

### 3.4 Computationally inferred immune-associated signatures of core genes

The consistent upregulation of the six glomerular core genes in DKD was observed across datasets. Single-gene GSEA displayed that their high expression was consistently and positively associated with upregulated immune and fibrotic pathways, including ECM receptor interaction, systemic lupus erythematosus, and the intestinal immune network for IgA production (**Supplementary Figures 5A–F**). Conversely, their expression was negatively associated with metabolic pathways, including branched-chain amino acid degradation, propanoate metabolism, and butanoate metabolism. This suggested that the glomerular core genes might be linked to the transition from metabolic homeostasis to immune inflammation and extracellular matrix remodeling.

A similar pattern was uncovered when single-gene GSEA was applied to tubulointerstitial core genes (**Supplementary Figures 5G–K**). In line with the box plot findings—downregulation of CA2, HSD11B2, and NQO2, and upregulation of MMP7 and CYP24A1 in DKD—these core genes were consistently associated with immune inflammation and metabolic suppression.

**Figure 4:**
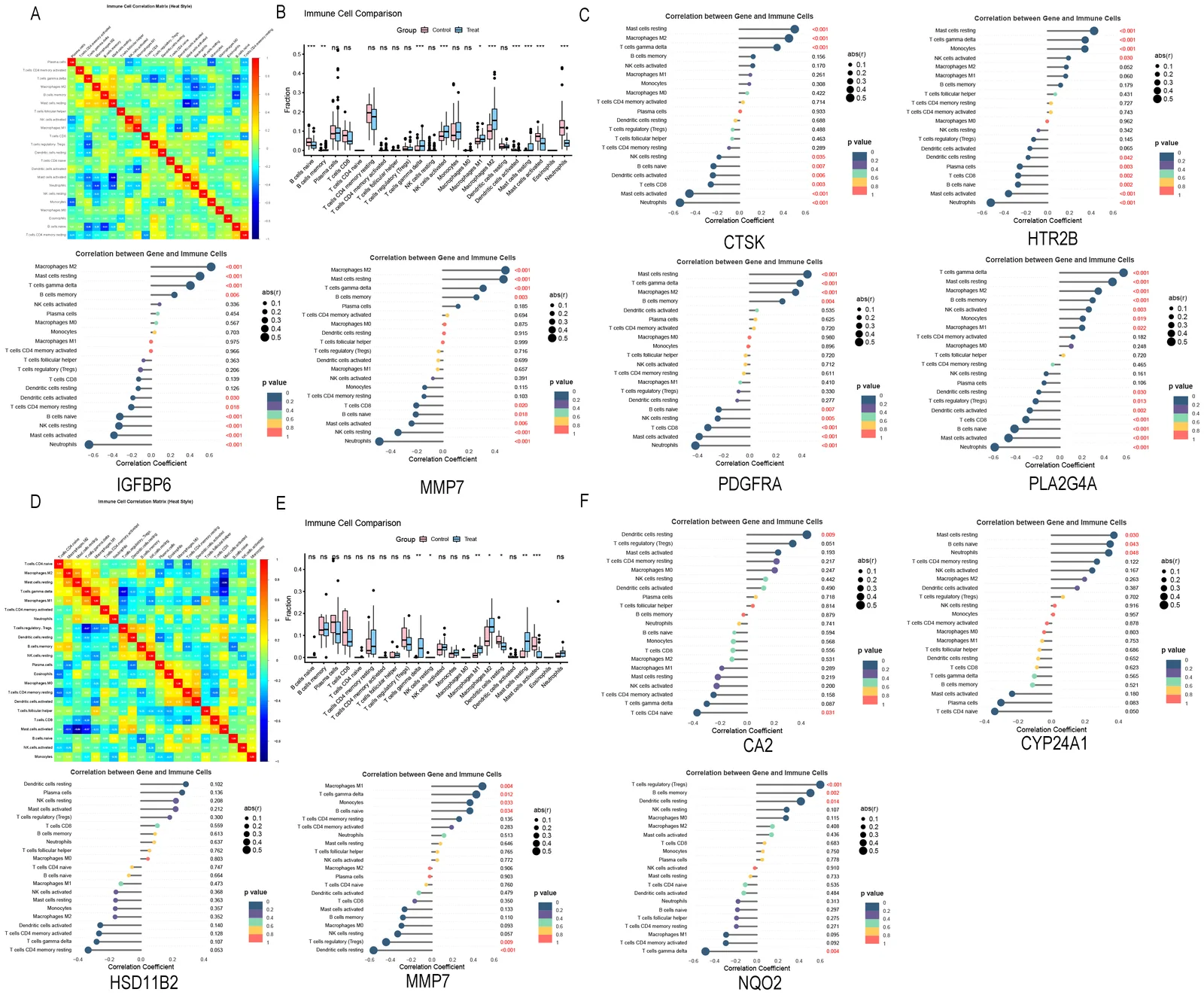
Association of core genes with immune cell infiltration in the DKD microenvironment. **(A)** Correlation analysis of 22 immune cell infiltration levels in the glomerular training set and **(D)** the tubulointerstitial training set. **(B)** Box plot comparing the infiltration fractions of 22 key immune cells in the glomerular training set and **(E)** the tubulointerstitial training set. **(C)** Lollipop plots illustrating the relationship between core genes and immune cells in the glomerular training set and **(F)** the tubulointerstitial training set. Red numbers indicate significant correlations, while black numbers indicate non-significant correlations. M2 macrophages and resting mast cells showed higher infiltration fractions in DKD samples compared to controls, while activated mast cells showed lower fractions; these infiltration patterns were correlated with core gene expression.

To investigate the immune roles of core genes, immune infiltration analysis was employed. In the DKD glomerular training set, memory B cells displayed a negative correlation with naïve B cells (r = -0.54) (**Figure 4A**). The infiltration levels of M2 macrophages, resting mast cells, and gamma-delta T cells were substantially upregulated, whereas naïve B cells, activated mast cells, and neutrophils were notably downregulated (**Figure 4B**). Core gene expression correlated positively with M2 macrophages, resting mast cells, and gamma-delta T cells, and negatively correlated with naïve B cells, activated mast cells, and neutrophils (**Figure 4C**). Given the observed upregulation of these core genes in DKD, these correlations are consistent with an association between core gene expression and the estimated immune composition; deconvolution-based estimates could not establish that the core genes regulate immune cell recruitment. Immune infiltration analysis in the validation and test sets further corroborated that elevated M2 macrophage infiltration and the phenotypic shift in mast cells—characterized by augmented resting and diminished activated populations co-varied with the expression of these core genes (**Supplementary Figure 6**).

In the DKD tubulointerstitial training set, resting mast cells exhibited a strong negative correlation with activated mast cells (r = -0.86) (**Figure 4D**). M2 macrophages, gamma-delta T cells, M1 macrophages, and resting mast cells were upregulated, while activated mast cells and resting dendritic cells were downregulated (**Figure 4E**). M1 macrophages, gamma-delta T cells, and resting mast cells displayed significant positive correlations with the core genes, while resting dendritic cells were negatively correlated (**Figure 4F**). Test set validation further reinforced the potential associations between these genes and the increased infiltration of resting mast cells, implicating this interaction in the regulation of DKD progression (**Supplementary Figure 7**).

Given the elevated M2 macrophage infiltration and the phenotypic shift in mast cells that occurred in DKD, GSVA was executed to quantitatively explore the relationship between core genes and macrophage- and mast cell-related biological pathways. Heatmaps revealed a consistent pattern: upregulated core genes were positively associated with enrichment scores of macrophage- and mast cell-related processes, whereas downregulated core genes were negatively associated (**Supplementary Figures 8A–F**, **S9A–E**). To precisely evaluate the correlation between gene expression and pathway enrichment scores, lollipop plots displayed the FDR-corrected associations, especially mast cell degranulation and M2 macrophage-related processes (**Supplementary Figures 8G–L**, **S9F–J**). Taken together, these computational analyses indicate that the expression of gut microbiota metabolite-related core genes co-varies with M2 macrophage- and mast cell-related signatures in the compartment-specific immune landscape of DKD. Because CIBERSORT and GSVA are inference-based methods applied to bulk transcriptomes, these results generate hypotheses about immune involvement but do not demonstrate that the core genes, or the candidate metabolites, modulate macrophage polarization or mast cell activity.

### 3.5 Single-cell analysis and correlation with renal function of core genes

Based on the KIT database, we utilized the GSE131882 (34) dataset to map cell type-specific gene expression profiles for the core genes, excluding *PDGFRA* (**Supplementary Figures 10A–D, F–JD**). Subsequently, we supplemented the analysis with single-nucleus RNA-seq (snRNA-seq) data from GSE195460 (28) to ascertain the expression of *PDGFRA* (**Supplementary Figure 10E**). Among the glomerular core genes, single-cell analysis unveiled cell-type-specific expression changes in DKD. Mesangial cells exhibited the highest activity, with *PLA2G4A* upregulated and both *IGFBP6* and *HTR2B* downregulated. *MMP7* was upregulated in the glomerular parietal epithelial cells and *PDGFRA* was expressed mainly in fibroblasts and mesangial cells. Conversely, the expression of *CTSK* presented no significant changes in glomerular-associated cells in the snRNA-seq dataset derived from early-stage DKD. Analysis of the tubulointerstitial core genes showed distinct expression patterns across different kidney segments in DKD. In the Proximal Convoluted Tubule (PCT), three genes were upregulated: *MMP7*, *CYP24A1*, and *NQO2*. In the Loop of Henle (LOH), expression of two genes was notably decreased: *HSD11B2* (despite being highly expressed across multiple cell types) and *NQO2*. In the Collecting Duct segments and the Distal Convoluted Tubule (DCT), *MMP7* and *CA2* experienced altered expression. Specifically, *MMP7* was upregulated in CD-PC, while *CA2* expression levels shifted significantly across the CD-PC, CD-ICA, and DCT.

We explored the relationship between each core gene and eGFR using the “Woroniecka Diabetes TubInt” and “Woroniecka Diabetes Glom” cohorts from the Nephroseq v5 database. In the glomerular cohort, all six core genes were negatively correlated with eGFR (**Supplementary Figures 11A–F**). After applying a Bonferroni-corrected threshold (P < 0.05/11) to account for multiple testing, except for *CTSK*, HTR2B and PDGFRA, these correlations were statistically significant. In the tubulointerstitial cohort, *CA2*, *HSD11B2*, and *NQO2* were positively correlated with eGFR, whereas *MMP7* and *CYP24A1* were negatively correlated (**Supplementary Figures 11G–K**). Except for CA2 (r=0.417, p=0.054), CYP24A1 (r=-0.463, p=0.03), and NQO2(r=0.475, p=0.025), these correlations were statistically significant. This aligns with our previous box plot findings (*CA2*, *HSD11B2*, and *NQO2* are downregulated in DKD, while *MMP7* and *CYP24A1* are upregulated). These clinical correlations indicate that dysregulation of these core genes is closely tied to the clinical deterioration of renal function in DKD patients.

### 3.6 The “M-S-M-T” as a hypothesis-generating framework

**Figure 5:**
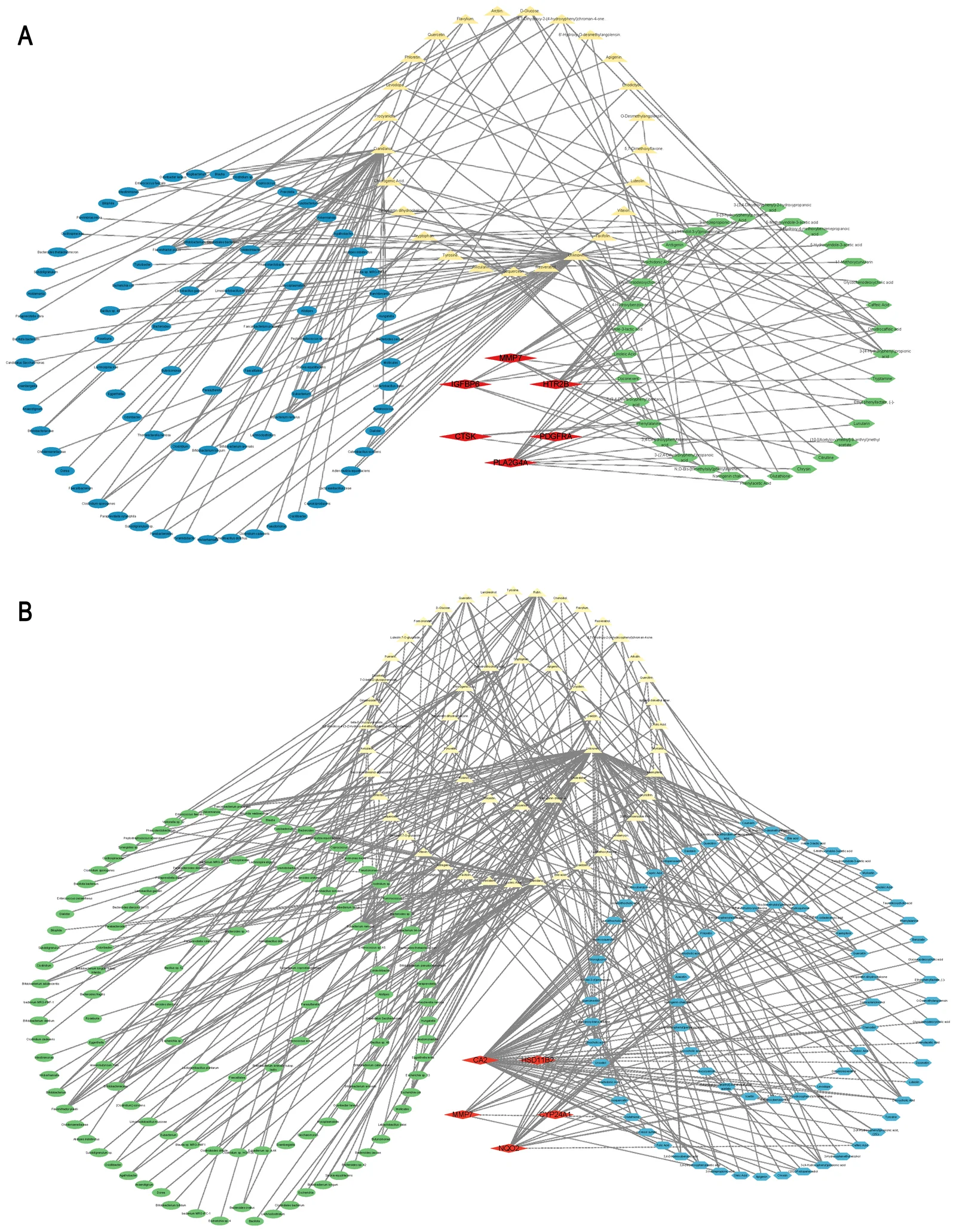
The “M-S-M-T” as a hypothesis-generating framework. **(A)** The “M-S-M-T” network constructed from glomerular core genes and **(B)** from tubulointerstitial core genes, visualizing potential gut–kidney interaction axes and illustrating the complex relationships among gut microbes, their metabolites, and host target genes.

Applying the core genes identified in the glomerular and tubulointerstitial datasets, we constructed “Microbiota–Substrate–Metabolite–Target” (“M-S-M-T”) networks to characterize the relationships among these four components. In the glomerular dataset, 79 gut microbiotas, 25 substrates, and 33 metabolites associated with the six core targets of DKD were illustrated. For the tubulointerstitial dataset, the network involved 116 gut microbiotas, 52 substrates, and 75 metabolites. These “M-S-M-T” networks were visualized by Cytoscape (v3.10.0) (35) (**Figure 5**). These networks are entirely prediction-based: they were assembled from curated microbe-substrate-metabolite-gene relationships and target prediction algorithms, and no microbiome sequencing, fecal or serum metabolomics, or renal metabolite measurement was performed in DKD patients or animal models.

### 3.7 The analysis of drug-likeness and toxicity

To screen for safe and potential therapeutic candidates targeting DKD, we evaluated the drug-likeness and toxicity of the metabolites. Focusing on the shared core gene, *MMP7*, the “M-S-M-T” network was traced back to corresponding metabolites. Preliminary screening based on Lipinski’s Rule of Five yielded eight candidate metabolites (**Supplementary Table 3**). However, all eight were subsequently excluded due to potential nephrotoxicity (Nephrotoxicity. DI > 0.3) or other organ toxicities (**Supplementary Table 4**). We broadened the search by assessing the “M-S-M-T” networks for the glomerular and tubulointerstitial datasets separately. Applying Lipinski’s rules again, we identified 33 (**Supplementary Table 5**) and 69 (**Supplementary Table 6**) drug-like metabolites for the glomerular and tubulointerstitial networks, respectively. Next, we used the ADMETlab 3.0 platform for toxicity prediction, converting the probability values into a scoring system: 0–0.1 (–), 0.1–0.3 (--), 0.7–0.9 (++), and 0.9–1.0 (+++) (36). Scores of (+++) or (++) indicate that a molecule is more likely to be toxic, while (-–) or (--) indicates relative safety or suitable properties. We further excluded metabolites with predicted nephrotoxicity, arrhythmia risk, or carcinogenicity (restricting the probability threshold at 0.3). This process screened five safe candidate metabolites for the glomerular network (**Table 2**) and six for the tubulointerstitial network. Caffeic acid and naringenin chalcone were carried forward because they were the only metabolites retained in both compartment-specific networks after drug-likeness and toxicity filtering, and were therefore the sole candidates with cross-compartment relevance. (**Supplementary Figure 12**).

**Table 2:** The evaluation of toxicity on key metabolites in datasets.

Dataset	Metabolite	Nephrotoxicity. DI	hERG	Carcinogenicity
glomerular	Dihydrocaffeic Acid	0.235651627	0.028109431	0.295978874
glomerular	3-(3,4-Dihydroxyphenyl)-2-hydroxypropanoic acid	0.10940969	0.049674861	0.176983878
glomerular	Caffeic Acid	0.125769675	0.037798077	0.178640023
glomerular	Naringenin chalcone	0.127120927	0.127702877	0.228894904
glomerular	5-(3,4-Dihydroxyphenyl) pentanoic acid	0.177659199	0.059462931	0.297561944
tubulointerstitial	Levodopa	0.142430261	0.076840147	0.109860674
tubulointerstitial	Leucocyanidin	0.037809175	0.087481752	0.28621009
tubulointerstitial	Caffeic acid	0.125769675	0.037798077	0.178640023
tubulointerstitial	Naringenin chalcone	0.127120927	0.127702877	0.228894904
tubulointerstitial	3,4-Dihydroxy-trans-stilbene	0.089576982	0.269619823	0.230938643

Nephrotoxicity. DI: Drug-induced nephrotoxicity.

### 3.8 Molecular docking for computational structural prioritization of compound–target interactions

**Figure 6:**
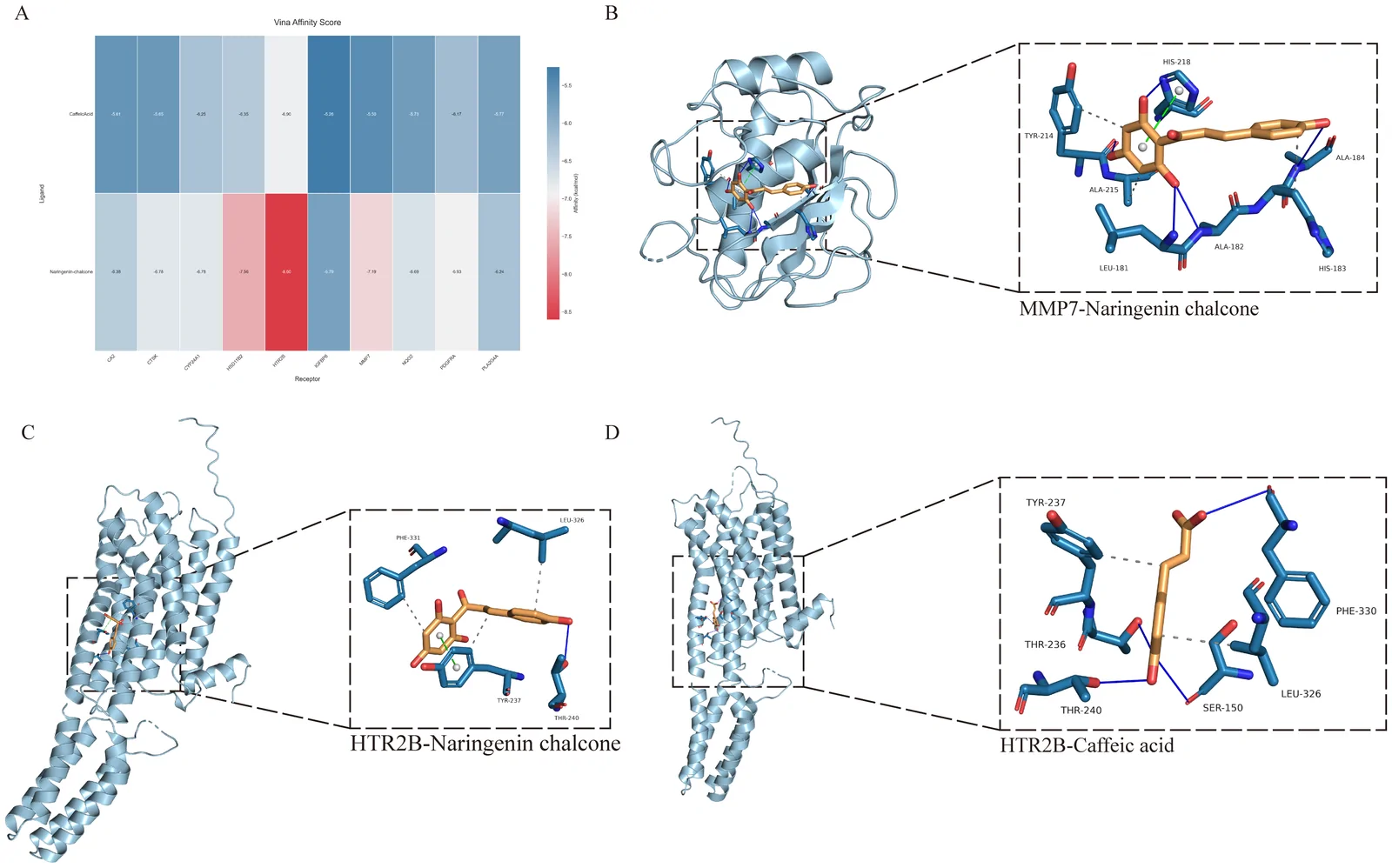
Molecular docking for computational structural prioritization of compound–target interactions. **(A)** Heatmap displaying the Vina affinity scores for molecular docking. The affinity scores are represented by the color gradient and the numerical values. **(B)** Docking pose of the MMP7–Naringenin chalcone complex, visualized via PLIP and PyMOL. **(C)** Docking pose of the HTR2B–Naringenin chalcone complex. **(D)** Docking pose of the HTR2B–Caffeic acid complex. These in silico predictions provided initial structural prioritization, with selected complexes subjected to molecular dynamics simulations for further stability assessment.

Molecular docking positioned naringenin chalcone as the superior candidate (**Figure 6A**). MMP7 was prioritized as the only core gene shared by the two compartments, and HTR2B as the glomerular target with the most favorable docking affinity, which defined the ligand-protein pairs taken forward for structural analysis. Naringenin chalcone exhibited robust affinity for HTR2B (-8.602 kcal/mol), surpassing the standard inhibitor SB-204741 (-8.027 kcal/mol) (**Supplementary Table 7**). Unlike SB-204741, which relied primarily on hydrophobic contacts (**Supplementary Table 8**), naringenin chalcone establishes a π-stacking interaction with TYR237 and dual hydrogen bonds with THR240 (**Figure 6C**, **Supplementary Table 9**). Caffeic acid similarly bound to HTR2B, albeit with lower affinity (-6.897 kcal/mol), utilizing a hydrophobic pocket anchored by TYR237 (**Figure 6D**, **Supplementary Table 10**). For MMP7, although naringenin chalcone showed lower affinity than the inhibitor Tanomastat (-8.081 kcal/mol) (**Supplementary Table 11**), it anchored to the active site with TYR214 and HIS183, and shared a key π-stacking interaction with HIS218 (**Figure 6B**, **Supplementary Table 12**). In silico results indicate structural capacity to occupy the binding pockets and that docking scores alone do not establish target engagement in a cellular context.

### 3.9 Molecular dynamics simulation of the MMP7- naringenin chalcone, HTR2B- naringenin chalcone, and HTR2B-caffeic acid complexes

**Figure 7:**
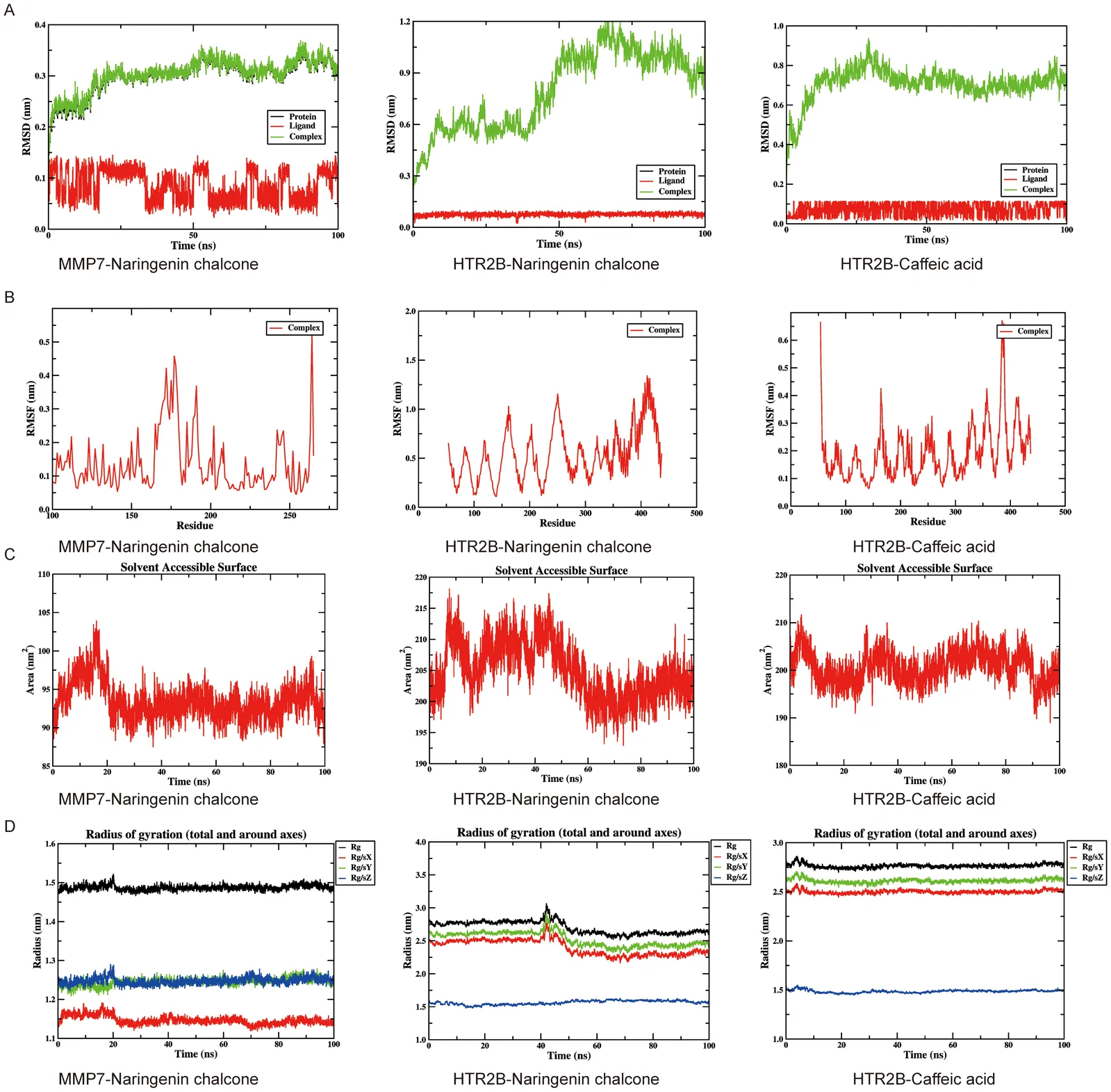
Molecular dynamics simulation of the MMP7- naringenin chalcone, HTR2B- naringenin chalcone, and HTR2B-caffeic acid complexes. **(A)** The RMSD curve. **(B)** The RMSF curve. **(C)** The SASA curve. **(D)** The Rg curve. The simulations supported the computational plausibility of the predicted interactions.

We executed MM/GBSA analysis on the three complexes. The calculated binding free energies (ΔG_bind_) for the HTR2B–naringenin chalcone and HTR2B–caffeic acid complexes were -108.1949 ± 11.6700 kJ/mol and -115.3688 ± 7.7848 kJ/mol, respectively, indicating greater stability than the MMP7–naringenin chalcone complex (-40.5371 ± 10.9060 kJ/mol) (**Supplementary Table 13**, **Supplementary Figures 13A–C**). Hydrogen bond mapping corroborated these findings, while the MMP7 complex formed 0–2 hydrogen bonds; both HTR2B complexes maintained 0–3 bonds, displaying a higher density and tighter distribution of interactions (**Supplementary Figures 13D–F**). The RMSD trajectories for the three complexes fluctuated tightly within 1.2 Å and showed no significant deviations, indicating high stability (**Figure 7A**). Higher RMSF values denote greater residue mobility, while lower values indicate restricted movement. The RMSF ranges were 0-4 Å for MMP7–naringenin chalcone, 0-10 Å for HTR2B–naringenin chalcone, and 0-4 Å for HTR2B–caffeic acid complexes (**Figure 7B**). All three complexes contained residues that exhibited high mobility. Structural compactness and solvent exposure, measured by Solvent Accessible Surface Area (SASA) and Radius of Gyration (Rg), remained stable across all systems, except for the transient Rg fluctuation in the MMP7 complex around 40 ns (**Figures 7C, D**). Finally, Free Energy Landscape (FEL) plots indicated that all complexes achieved thermodynamic equilibrium, with the HTR2B complexes exhibiting multiple metastable states within the low-energy basins (**Supplementary Figures 13G–L**).

### 3.10 *In vitro* evaluation of the therapeutic effects and target dependency of naringenin chalcone and caffeic acid

**Figure 8:**
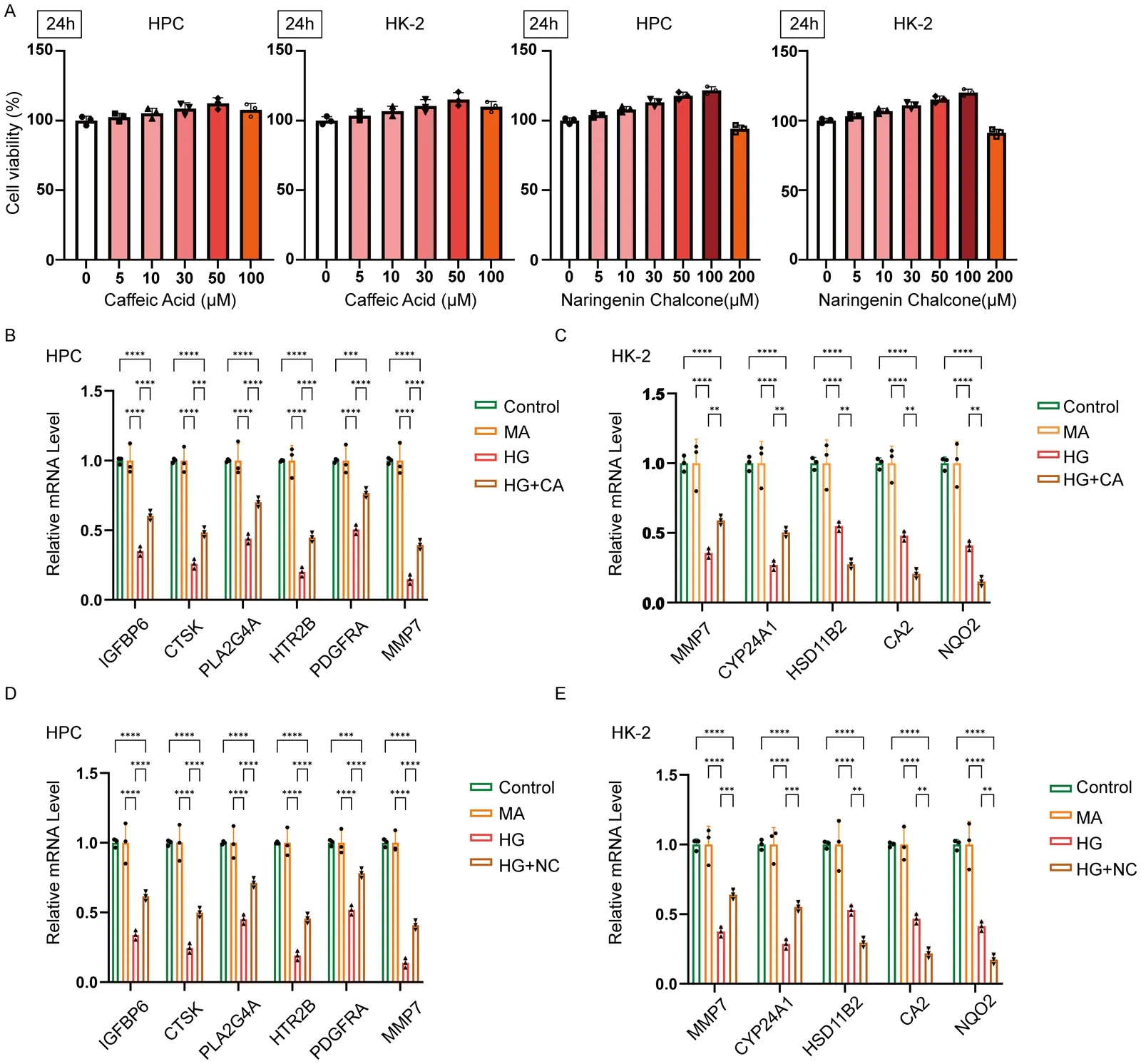
*In vitro* evaluation of the therapeutic effects and target dependency of naringenin chalcone and caffeic acid. **(A)** CCK-8 assays of cell viability after drug treatment. **(B–E)** RT-qPCR analysis in podocytes **(B, D)** and HK-2 cells **(C, E)**, displaying the effect of naringenin chalcone and caffeic acid. These results provided preliminary functional support at the mRNA and protein levels. (n = 3).

HK-2 cells and podocytes were cultured to explore the potential modulatory effects of naringenin chalcone and caffeic acid. In HG-induced HK-2 cells, MMP7, CYP24A1, HSD11B2, CA2, and NQO2 were downregulated. HG exposure in podocytes triggered the downregulation of IGFBP6, CTSK, PLA2G4A, HTR2B, PDGFRA, and MMP7. Based on CCK-8 results (**Figure 8A**), the working concentration of naringenin chalcone was set at 100 μM and caffeic acid at 50 μM for subsequent experiments. Naringenin chalcone or caffeic acid rescued HG-induced downregulation of core genes in podocytes (**Figures 8B, D**). In HK-2 cells, HG-induced dysregulation of core genes was reversed by naringenin chalcone or caffeic acid (**Figures 8C, E**). Western blot analysis revealed that HG treatment significantly downregulated MMP-7 protein expression in both HK-2 cells and podocytes, as well as HTR2B in podocytes, compared with the control and mannitol groups. Both metabolites effectively reversed these changes (**Supplementary Figure 14**). To further investigate the potential anti-apoptotic protective effects of the drugs, Bcl-2 expression was examined in both cell types. HG treatment markedly downregulated Bcl-2 compared with the control and mannitol groups, whereas treatment with naringenin chalcone or caffeic acid restored its expression (**Supplementary Figure 14**). To explore the target-dependent effects of the two drugs in both cell types, a selective HTR2B inhibitor RS-127445 and an MMP-7 inhibitor marimastat were respectively added to the culture. These inhibitors not only exacerbated HG-induced Bcl-2 downregulation but also abolished the restorative effect of the drugs on Bcl-2 expression (**Supplementary Figure 15**). Taken together, the anti-apoptotic protective effects were attenuated by blockade of HTR2B or MMP-7, suggesting these targets contribute to the observed effect.

## 4 Discussion

The gut-kidney axis has emerged as a critical area of investigation regarding the pathogenesis of DKD (37–39). However, current research often treats the kidney as a homogeneous organ, obscuring the distinct molecular landscapes of the glomerular and tubulointerstitial compartments. In this study, we addressed this limitation by constructing a compartment-specific “M-S-M-T” regulatory network. By integrating machine learning, MR, clinical database, single-cell analysis, and *in vitro* experimental validation, we identified compartment-specific core genes driven by gut microbiota metabolites and prioritized caffeic acid and naringenin chalcone as shared candidate metabolites, for which a preliminary transcriptional response was observed *in vitro*. In the following discussion, we deliberately distinguish four levels of evidence: computational prediction, external database corroboration, molecular simulation, and experiment.

Employing machine learning, accompanied by clinical eGFR correlation, MR, and transcriptomic profiling, specific core genes were identified for the glomerulus (*IGFBP6*, *PLA2G4A*, *CTSK*, *HTR2B*, *PDGFRA*, *MMP7*) and the tubulointerstitium (*CA2*, *HSD11B2*, *NQO2*, *MMP7*, *CYP24A1*). The shared target *MMP7* was upregulated consistently in both compartments and negatively related to eGFR. However, its pQTL was associated with preserved eGFR, possibly attributable to histologic origin. Previous literature indicates that *MMP7* expression is linked to interstitial fibrosis and predicts accelerated eGFR decline (40–42). In the glomerulus, SHAP analysis indicated that *IGFBP6* and *CTSK* served as superior diagnostic contributors. *CTSK*, *HTR2B*, and *PLA2G4A* were upregulated in DKD, negatively associated with eGFR, with respective QTLs causally linked to DKD-related renal outcomes via MR. Current evidence shows that PLA2G4A is a key node in programmed cell death pathways in DKD (43), and HTR2B is associated with autophagy-mediated DKD tubulointerstitial injury (44). In the tubulointerstitium, the dependence plot displayed that the co-upregulation of *MMP7* and *CYP24A1* served as high-risk signals for DKD prediction. The expression of *CYP24A1* was high and negatively associated with eGFR in DKD. Previous studies indicated that renal *CYP24A1* is upregulated in experimental diabetic animal models, with caspase-3-mediated tubular apoptosis. Vitamin D supplementation or NF-κB inhibition downregulates *CYP24A1* and ameliorates DKD (45, 46). Interestingly, predicted high expression of its eQTL was linked to decreased DKD risk, possibly because its eQTL originates from blood, and its function is tissue-specific. In contrast, *HSD11B2* was downregulated and positively correlated with eGFR, while its mQTL preserved eGFR. Loss of HSD11B2 expression in mesangial and endothelial cells promotes fibrosis and renal injury in DKD by agonizing the mineralocorticoid receptor (47, 48).

The largely non-overlapping composition of the two signatures is biologically coherent once the distinct microenvironments of the two compartments are considered. The glomerulus is dominated by hemodynamic and mechanical stress: hyperglycemia-induced afferent arteriolar vasodilation produces capillary hyperfiltration, so that podocytes are continuously exposed to elevated mechanical strain, while basement membrane degradation and mesangial matrix deposition further aggravate filtration barrier dysfunction. Consistent with this, activation of podocyte TRPC6 upregulates MMP7 (49), which in turn degrades the slit diaphragm protein nephrin and compromises barrier integrity (50); stretch also induces CTSK (51), and CTSK deletion attenuates glomerulosclerosis and foot process effacement (52); mechanical stretch directly phosphorylates and activates PLA2G4A (53), which participates in TNF-alpha-driven mesangial inflammation (54) and thereby converts a mechanical signal into an inflammatory one; and PDGFRA, a marker of mesangial cell precursors (55), drives fibroblast-to-myofibroblast differentiation and extracellular matrix synthesis through the TGFB1/SMAD pathway (56, 57). The glomerular core genes can thus be read as a continuous mechanosensing-protease activation-inflammation-fibrosis cascade.

In the tubulointerstitium the dominant physiology is reabsorption and acid-base handling. MMP7 indirectly degrades collagen IV, laminin and heparan sulphate proteoglycans of the tubular basement membrane through activation of MMP2 and MMP9 (58), thereby undermining the structural basis of reabsorption (59). In the proximal tubule, FGF23 activates CYP24A1 transcription and accelerates vitamin D catabolism (60), and reduced vitamin D availability limits tubular calcium reabsorption. CA2 is a core enzyme of tubular reabsorption that couples to NHE3 to drive sodium and bicarbonate reabsorption (61), and its deficiency impairs both proximal bicarbonate reabsorption and distal urinary acidification, producing mixed renal tubular acidosis (62). TNF-alpha directly represses HSD11B2 transcription through NF-kappaB and Egr-1 (63), and loss of HSD11B2 permits inappropriate cortisol activation of the mineralocorticoid receptor, increasing distal sodium reabsorption and potassium excretion (64). The compartment-specific signatures therefore mirror compartment-specific physiology: mechanical and matrix biology in the glomerulus, and transport and mineral-electrolyte biology in the tubulointerstitium, with MMP7 serving as the shared node of basement membrane remodeling in both.

Crucially, GSEA implied that core genes participated in systemic immune pathways jointly. Immune cell infiltration analysis and GSVA of core genes indicated a higher estimated M2 macrophage fraction and an activated-to-resting shift of the estimated mast cell composition in both compartments. In the tubulointerstitium, MMP7 emerged as the primary SHAP contributor. M2 macrophages release TGF-β to aggravate renal fibrosis and suppress immune reaction in DKD. The miR-411-3p/MMP7 axis critically regulates this process, as *MMP7* upregulation directly induces M2 polarization (65). The synergistic effect between *CYP24A1* and *MMP7* can be partially explained by immune functions. CYP24A1 promotes M2 macrophage polarization by degrading vitamin D (66). Their upregulation may collectively promote M2 macrophage polarization and fibrosis in DKD. In the glomerulus, the top SHAP contributors, *IGFBP6* and *CTSK*, played critical immune roles. *IGFBP6* serves as a suppressor of macrophage chemotaxis via suppressing *CCL2* transcription (67), while *CTSK* induces M2 macrophage polarization via TLR4/mTOR signaling (68). Mast cells infiltrate more into the kidney and undergo degranulation (69), releasing chymase to promote renal fibrosis as DKD progresses (70), aligning with our GSVA results. We therefore propose, as a hypothesis rather than as a demonstrated mechanism, that gut microbiota metabolites may act as systemic signaling molecules that perturb these core genes and thereby contribute to the pro-fibrotic immune microenvironment of DKD. Because our immune findings rest entirely on deconvolution and gene set scoring, this hypothesis requires validation with macrophage and mast cell markers, as well as co-culture or conditioned-medium experiments before any regulatory conclusion can be drawn.

Our predicted relationships can be compared with recently reported clinical multi-omics profiles of the gut-kidney axis in DKD (71). At the taxonomic level, the overlap is partial: Bacteroides appears in both compartment networks, whereas Klebsiella, Faecalibaculum and Dubosiella do not. Nevertheless, the overall architecture is concordant, as metabolites predicted to target our core genes derive both from opportunistic taxa (e.g., Bacteroides, Escherichia coli, Clostridium) and from SCFA-producing commensals (e.g., Faecalibacterium, Roseburia, Akkermansia). At the pathway level, branched-chain amino acid degradation, significantly suppressed in clinical DKD by KEGG enrichment, was also negatively enriched in all six glomerular core gene high-expression groups. At the metabolite level, our networks independently recovered six bile acid-related metabolites together with a broad set of phenylalanine, tyrosine and tryptophan derivatives acting on MMP7, CTSK, CA2, HSD11B2 and HTR2B, thereby providing compartment-resolved candidate targets for the bile acid and aromatic amino acid alterations reported in patient cohorts. At the immune level, the reported rise in circulating lipopolysaccharide is consistent with our observation that metabolites of lipopolysaccharide-associated opportunistic taxa are predicted to target MMP7, CA2 and HSD11B2. These convergences are encouraging but remain correlative: the present study contains no microbiome sequencing or metabolomic measurement of its own, so this comparison supports the plausibility of the “M-S-M-T” framework rather than validating it.

To explore the gut-kidney axis, “M-S-M-T” networks were constructed on the basis of compartment-specific core genes. Via drug-like analysis and toxic analysis, 9 safe gut microbiota metabolites that targeted core genes and possessed druggability were screened. Among them, the shared molecules caffeic acid and naringenin chalcone were evaluated for their binding affinity with core genes through molecular docking and molecular dynamics simulations. Furthermore, *in vitro* experiments showed that both metabolites normalized HG-induced transcriptional changes in the core genes, which is a preliminary observation rather than proof of therapeutic efficacy. Prior studies exhibit that caffeic acid and its derivatives exhibit significant anti-inflammatory and antioxidant effects (72) and are capable of reversing the oxidative stress status observed in DKD (73). Furthermore, caffeic acid affords protection by modulating the autophagy pathway (74). As the metabolic precursor of caffeic acid, Chlorogenic acid ameliorates renal fibrosis and lipid accumulation in DKD mice by suppressing the Notch1 and Stat3 signaling pathways (75). Naringenin chalcone, a member of the chalcone family, possesses marked anti-inflammatory activity (76).

The translational distance between these candidates and clinical practice should be stated explicitly. Among all core genes, only MMP7 currently carries multi-dimensional clinical evidence as a measurable biomarker: unbiased kidney tissue proteomics in cohorts including DKD patients identified plasma MMP7 as a predictor of subsequent renal function decline and linked tubular MMP7 to the degree of fibrosis and to eGFR (40); urinary MMP7 has been associated with disease progression and mortality in type 2 diabetes (77); and MMP7 in urinary extracellular vesicles outperforms whole-urine measurement for identifying rapid progressors in autosomal dominant polycystic kidney disease (78), which suggests that urinary extracellular vesicles are a promising matrix for future DKD studies. PDGFRA occupies an intermediate position: circulating detection is difficult, but its specific enrichment in activated (Tcf21-positive) kidney fibroblasts (57) makes it a useful tissue marker of fibrosis in biopsy sections rather than a liquid-biopsy analyte. The remaining core genes have not been evaluated in any clinical cohort and are at present suitable only for mechanistic discussion. On the therapeutic side, no target-directed clinical trial exists for any of these genes. PDGFRA can be addressed by multi-target tyrosine kinase inhibitors such as imatinib and sunitinib, with supporting animal evidence in renal fibrosis (79); MMP7 by broad-spectrum and selective MMP inhibitors with preclinical antifibrotic activity (80); and CA2 by the approved carbonic anhydrase inhibitor acetazolamide, which nevertheless reduced glomerular filtration in obese non-diabetic individuals (81) and thereby illustrates the risk of interfering with a physiologically essential transport enzyme. The two candidate metabolites carry their own liabilities: both have limited oral bioavailability and chemical stability (82, 83), naringenin chalcone has been studied predominantly in plant science, and caffeic acid is approved in China only for thrombocytopenia (84). Caffeic acid is further reduced by the gut microbiota to dihydrocaffeic acid, itself proposed as a risk marker for type 2 diabetes, so that microbial metabolism may either attenuate the parent compound or generate informative biomarkers (85). Nanoparticle delivery has improved bioavailability and efficacy in preclinical work (86), and meta-analyses of caffeic acid-containing traditional Chinese medicine formulations in chronic kidney disease report modest benefit (87); structural modification, optimization of the route of administration and dedicated renal clinical studies would nevertheless all be required before translation.

While this study proposes a compartment-resolved framework linking the gut microbiota to the glomerular and tubulointerstitial compartments in DKD, several limitations should be acknowledged. MR analysis was based on blood-sourced genetic instruments from European populations, which limits both tissue relevance and transferability to other ancestries; MR results are therefore presented as supportive genetic evidence rather than causal validation, and single-SNP instruments could not be subjected to pleiotropy testing. Second, the M-S-M-T networks are predictions derived from curated databases and target prediction algorithms; we performed no microbiome sequencing and no fecal, serum or renal metabolomics, and we therefore cannot demonstrate that the predicted metabolites are altered in DKD or that they reach the renal compartments at biologically relevant concentrations. Third, the immune findings derive from deconvolution and gene set scoring of bulk transcriptomes and are correlative. Fourth, the *in vitro* data are limited to two cell lines. Additional validation in primary renal cells or animal models, along with siRNA-based target-dependency experiments across a broader range of functional readouts, will be required to fully establish the mechanistic roles of these targets. Fifth, several core genes did not reach statistical significance in individual validation or test cohorts, most plausibly because of small sample sizes and clinical heterogeneity. Additionally, the *in vitro* experiments used acute high-glucose exposure in two cell lines, which cannot fully reproduce the chronic DKD microenvironment or the cellular complexity of kidney tissue, and this may contribute to directional discrepancies between the cell-based assays and the bulk transcriptomic data. Finally, the least-significant-difference *post hoc* test used for the cell experiments is less conservative than Tukey, Bonferroni or Dunnett procedures, although all comparisons were pre-specified and every experiment was independently repeated. Accordingly, the “M-S-M-T” network is presented as a hypothesis-generating framework rather than as a validated regulatory axis. Future research should test it in real-world clinical samples with gut microbiome sequencing and with direct measurement of caffeic acid and naringenin chalcone in serum, feces, urine and kidney tissue, before intervention strategies targeting specific microbial components are pursued.

## 5 Conclusion

In this study, core genes specific to the glomerulus (*IGFBP6*, *PLA2G4A*, *CTSK*, *HTR2B*, *PDGFRA*, *MMP7*) and tubulointerstitium (*CA2*, *HSD11B2*, *NQO2*, *MMP7*, *CYP24A1*) were identified. Further, MR, immune infiltration analysis, single-cell transcriptomics, and a clinical cohort were used to explore the role and mechanism of core genes in DKD. Moreover, a corresponding “M-S-M-T” network was constructed for each compartment. Furthermore, molecular docking, molecular dynamics simulations and *in vitro* experiments prioritized caffeic acid and naringenin chalcone as shared candidate metabolites. These findings establish a hypothesis-generating framework for the gut-kidney axis in DKD and define specific priorities for validation in clinical microbiome and metabolomic studies.

## Data Availability

The datasets presented in this study can be found in online repositories. The names of the repository/repositories and accession number(s) can be found in the article/**Supplementary Material**.
