# Supplementary material for: Integrative multi-omics analysis prioritizes compartment-specific candidate targets of gut microbiota metabolites in diabetic kidney disease
Source: Front Immunol. 2026 Aug 31;17:1925910. doi: 10.3389/fimmu.2026.1925910 (PMC13583601; doi:10.3389/fimmu.2026.1925910)
Supplement: Supplementary file 1 [file DataSheet1.zip › Supplementary Figures/figureS1.pdf]

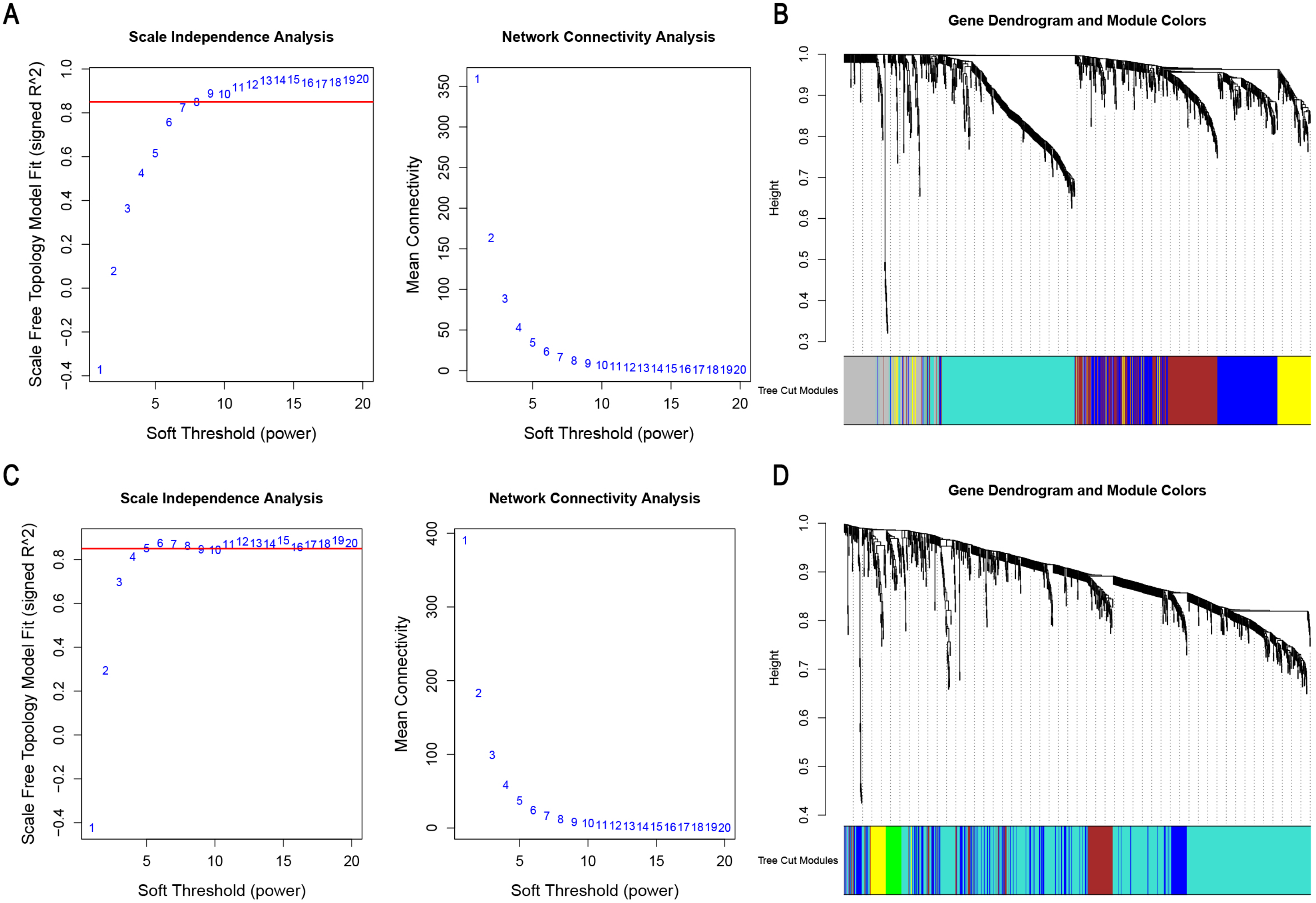
A
Scale Independence Analysis
Scale Free Topology Model Fit (signed R^2)
Soft Threshold (power)
Network Connectivity Analysis
Mean Connectivity
Soft Threshold (power)
B
Gene Dendrogram and Module Colors
Height
Tree Cut Modules
C
Scale Independence Analysis
Scale Free Topology Model Fit (signed R^2)
Soft Threshold (power)
Network Connectivity Analysis
Mean Connectivity
Soft Threshold (power)
D
Gene Dendrogram and Module Colors
Height
Tree Cut Modules
