# Supplementary material for: Integrative multi-omics analysis prioritizes compartment-specific candidate targets of gut microbiota metabolites in diabetic kidney disease
Source: Front Immunol. 2026 Aug 31;17:1925910. doi: 10.3389/fimmu.2026.1925910 (PMC13583601; doi:10.3389/fimmu.2026.1925910)
Supplement: Supplementary file 1 [file DataSheet1.zip › Supplementary Figures/figureS10.pdf]

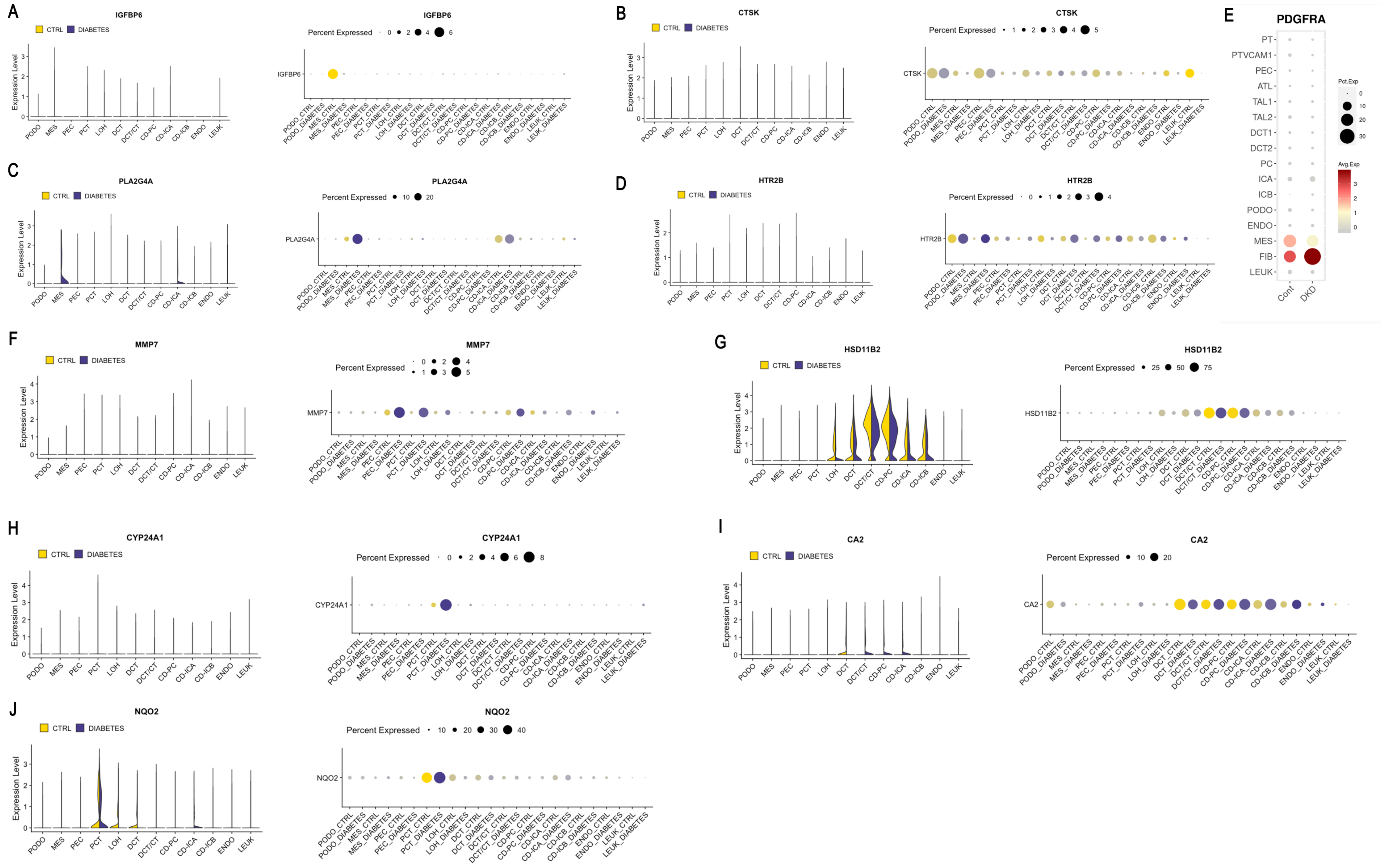
A
IGFBP6
CTRL DIABETES
Percent Expressed 0 2 4 6
B
CTSK
Percent Expressed 1 2 3 4 5
E
PDGFRA
PT
PTVCAM1
PEC
ATL
TAL1
TAL2
DCT1
DCT2
PC
ICA
ICB
PODO
ENDO
MES
FIB
LEUK
Cont
DKD
Pct.Exp
Avg.Exp
C
PLA2G4A
Percent Expressed 10 20
D
HTR2B
Percent Expressed 0 1 2 3 4
F
MMP7
Percent Expressed 0 2 4 1 3 5
G
HSD11B2
Percent Expressed 25 50 75
H
CYP24A1
Percent Expressed 0 2 4 6 8
I
CA2
Percent Expressed 10 20
J
NQO2
Percent Expressed 10 20 30 40
Expression Level
PODO
MES
PEC
PCT
LOH
DCT
DCT/CT
CD-PC
CD-ICA
CD-ICB
ENDO
LEUK
