# Supplementary material for: Integrative multi-omics analysis prioritizes compartment-specific candidate targets of gut microbiota metabolites in diabetic kidney disease
Source: Front Immunol. 2026 Aug 31;17:1925910. doi: 10.3389/fimmu.2026.1925910 (PMC13583601; doi:10.3389/fimmu.2026.1925910)
Supplement: Supplementary file 1 [file DataSheet1.zip › Supplementary Figures/figureS11.pdf]

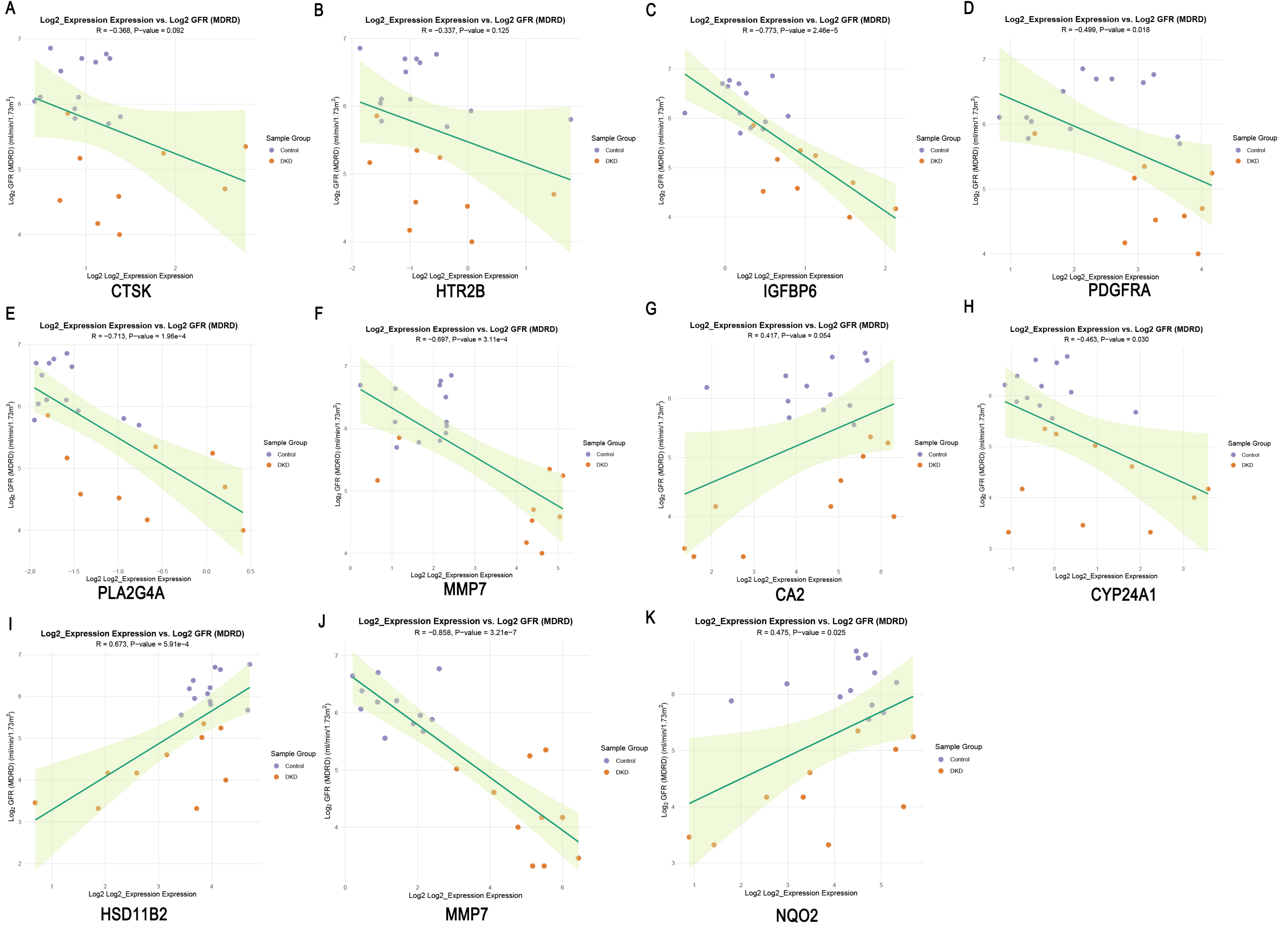
A
Log2_Expression Expression vs. Log2 GFR (MDRD)
R = −0.368, P-value = 0.092
CTSK
B
Log2_Expression Expression vs. Log2 GFR (MDRD)
R = −0.337, P-value = 0.125
HTR2B
C
Log2_Expression Expression vs. Log2 GFR (MDRD)
R = −0.773, P-value = 2.46e−5
IGFBP6
D
Log2_Expression Expression vs. Log2 GFR (MDRD)
R = −0.499, P-value = 0.018
PDGFRA
E
Log2_Expression Expression vs. Log2 GFR (MDRD)
R = −0.713, P-value = 1.96e−4
PLA2G4A
F
Log2_Expression Expression vs. Log2 GFR (MDRD)
R = −0.697, P-value = 3.11e−4
MMP7
G
Log2_Expression Expression vs. Log2 GFR (MDRD)
R = 0.417, P-value = 0.054
CA2
H
Log2_Expression Expression vs. Log2 GFR (MDRD)
R = −0.463, P-value = 0.030
CYP24A1
I
Log2_Expression Expression vs. Log2 GFR (MDRD)
R = 0.673, P-value = 5.91e−4
HSD11B2
J
Log2_Expression Expression vs. Log2 GFR (MDRD)
R = −0.858, P-value = 3.21e−7
MMP7
K
Log2_Expression Expression vs. Log2 GFR (MDRD)
R = 0.475, P-value = 0.025
NQO2
Log2 Log2_Expression Expression
Log2 GFR (MDRD) (ml/min/1.73m2)
Sample Group
Control
DKD
