# Supplementary material for: Integrative multi-omics analysis prioritizes compartment-specific candidate targets of gut microbiota metabolites in diabetic kidney disease
Source: Front Immunol. 2026 Aug 31;17:1925910. doi: 10.3389/fimmu.2026.1925910 (PMC13583601; doi:10.3389/fimmu.2026.1925910)
Supplement: Supplementary file 1 [file DataSheet1.zip › Supplementary Figures/figureS13.pdf]

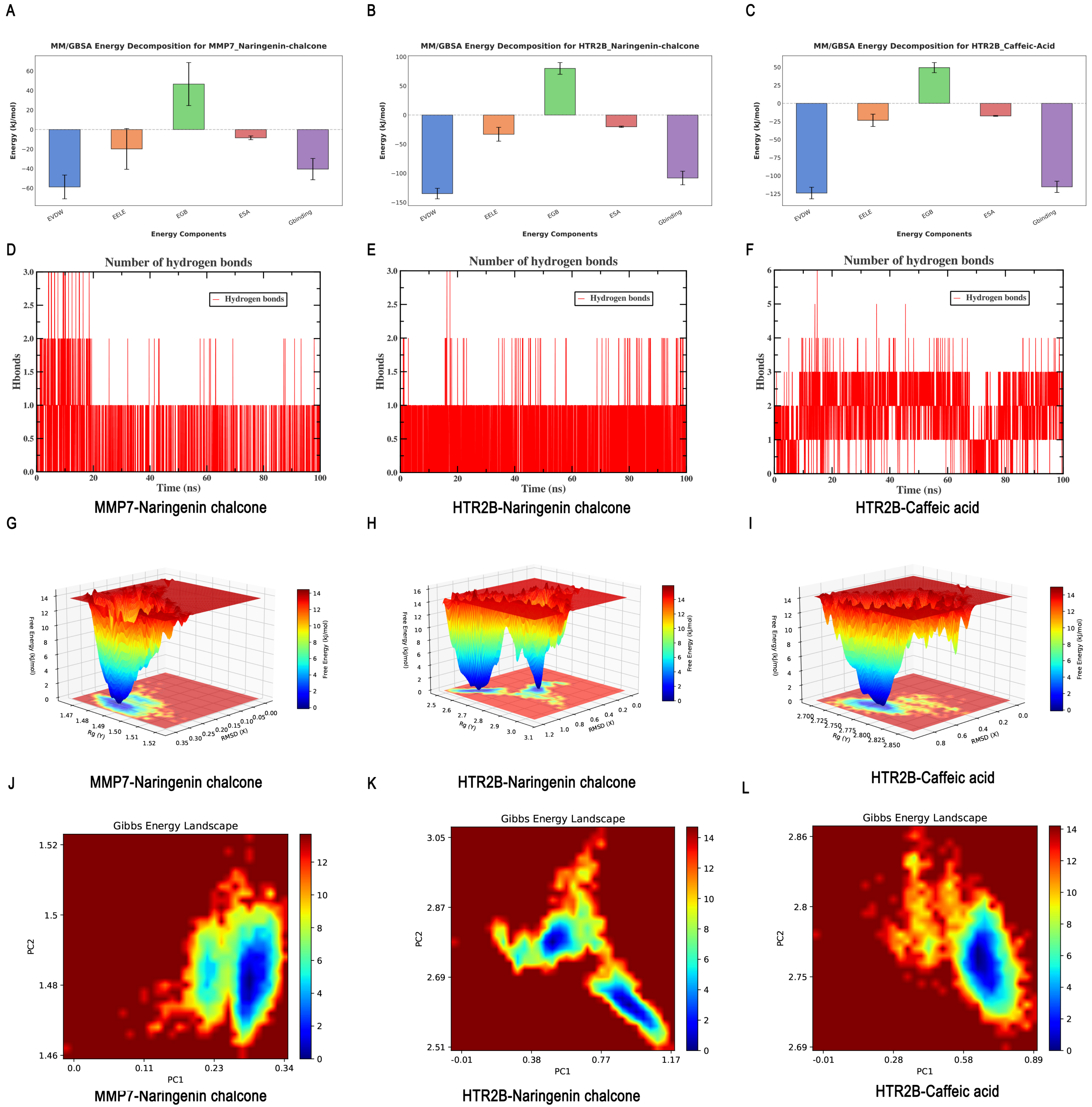
A
MM/GBSA Energy Decomposition for MMP7_Naringenin-chalcone
Energy (kJ/mol)
EVDW
EELE
EGB
ESA
Gbinding
Energy Components
B
MM/GBSA Energy Decomposition for HTR2B_Naringenin-chalcone
Energy (kJ/mol)
EVDW
EELE
EGB
ESA
Gbinding
Energy Components
C
MM/GBSA Energy Decomposition for HTR2B_Caffeic-Acid
Energy (kJ/mol)
EVDW
EELE
EGB
ESA
Gbinding
Energy Components
D
Number of hydrogen bonds
Hydrogen bonds
Hbonds
Time (ns)
MMP7-Naringenin chalcone
E
Number of hydrogen bonds
Hydrogen bonds
Hbonds
Time (ns)
HTR2B-Naringenin chalcone
F
Number of hydrogen bonds
Hydrogen bonds
Hbonds
Time (ns)
HTR2B-Caffeic acid
G
Free Energy (kJ/mol)
Rg (Y)
RMSD (X)
H
Free Energy (kJ/mol)
Rg (Y)
RMSD (X)
I
Free Energy (kJ/mol)
Rg (Y)
RMSD (X)
J
MMP7-Naringenin chalcone
K
HTR2B-Naringenin chalcone
HTR2B-Caffeic acid
L
Gibbs Energy Landscape
PC2
PC1
MMP7-Naringenin chalcone
Gibbs Energy Landscape
PC2
PC1
HTR2B-Naringenin chalcone
Gibbs Energy Landscape
PC2
PC1
HTR2B-Caffeic acid
