# Supplementary material for: Integrative multi-omics analysis prioritizes compartment-specific candidate targets of gut microbiota metabolites in diabetic kidney disease
Source: Front Immunol. 2026 Aug 31;17:1925910. doi: 10.3389/fimmu.2026.1925910 (PMC13583601; doi:10.3389/fimmu.2026.1925910)
Supplement: Supplementary file 1 [file DataSheet1.zip › Supplementary Figures/figureS14.pdf]

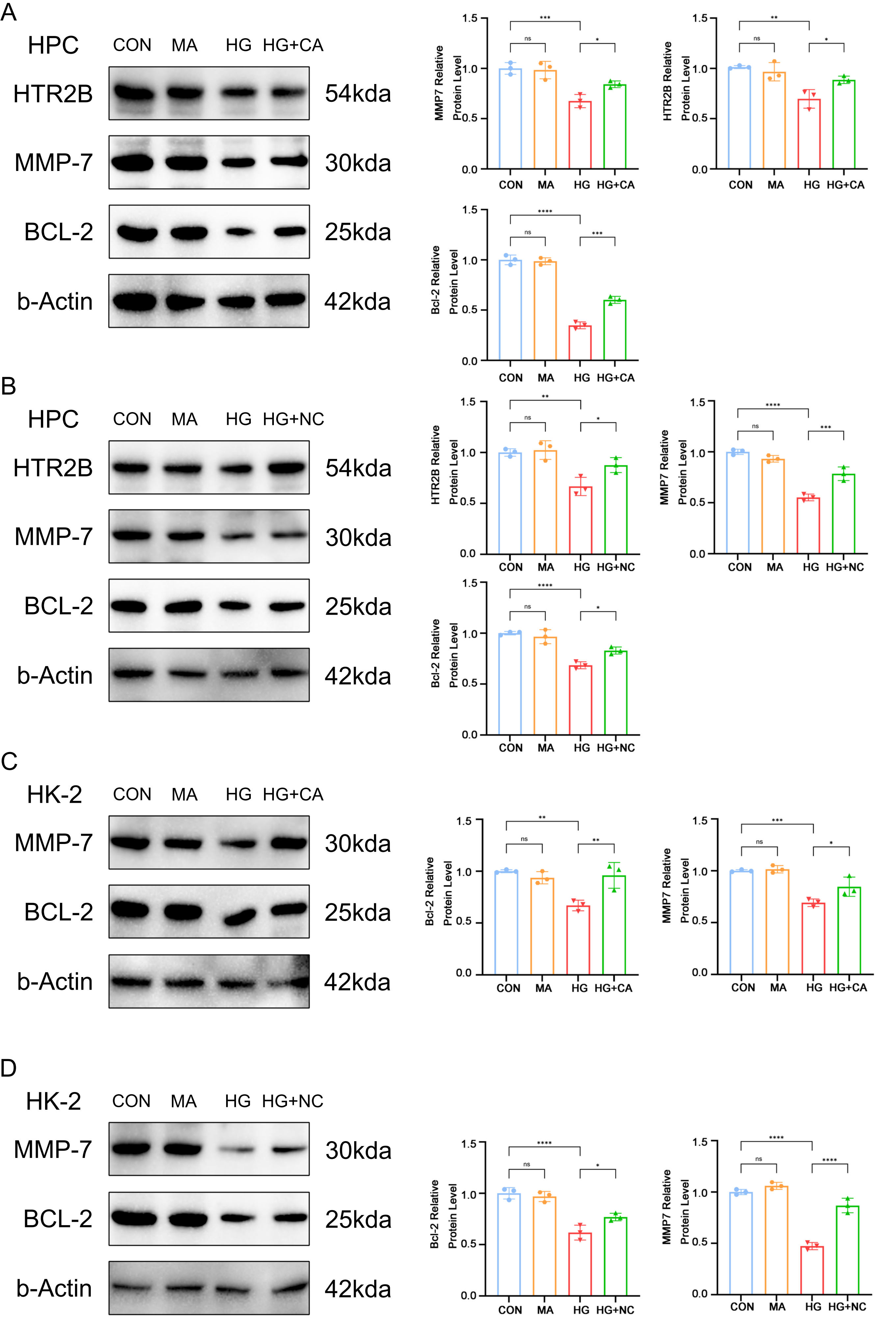
A
HPC
CON MA HG HG+CA
HTR2B
54kda
MMP-7
30kda
BCL-2
25kda
b-Actin
42kda
MMP7 Relative Protein Level
HTR2B Relative Protein Level
Bcl-2 Relative Protein Level
ns
*
**
***
****
B
HPC
CON MA HG HG+NC
HTR2B
54kda
MMP-7
30kda
BCL-2
25kda
b-Actin
42kda
HTR2B Relative Protein Level
MMP7 Relative Protein Level
Bcl-2 Relative Protein Level
C
HK-2
CON MA HG HG+CA
MMP-7
30kda
BCL-2
25kda
b-Actin
42kda
Bcl-2 Relative Protein Level
MMP7 Relative Protein Level
D
HK-2
CON MA HG HG+NC
MMP-7
30kda
BCL-2
25kda
b-Actin
42kda
Bcl-2 Relative Protein Level
MMP7 Relative Protein Level
