# Supplementary material for: Integrative multi-omics analysis prioritizes compartment-specific candidate targets of gut microbiota metabolites in diabetic kidney disease
Source: Front Immunol. 2026 Aug 31;17:1925910. doi: 10.3389/fimmu.2026.1925910 (PMC13583601; doi:10.3389/fimmu.2026.1925910)
Supplement: Supplementary file 1 [file DataSheet1.zip › Supplementary Figures/figureS15.pdf]

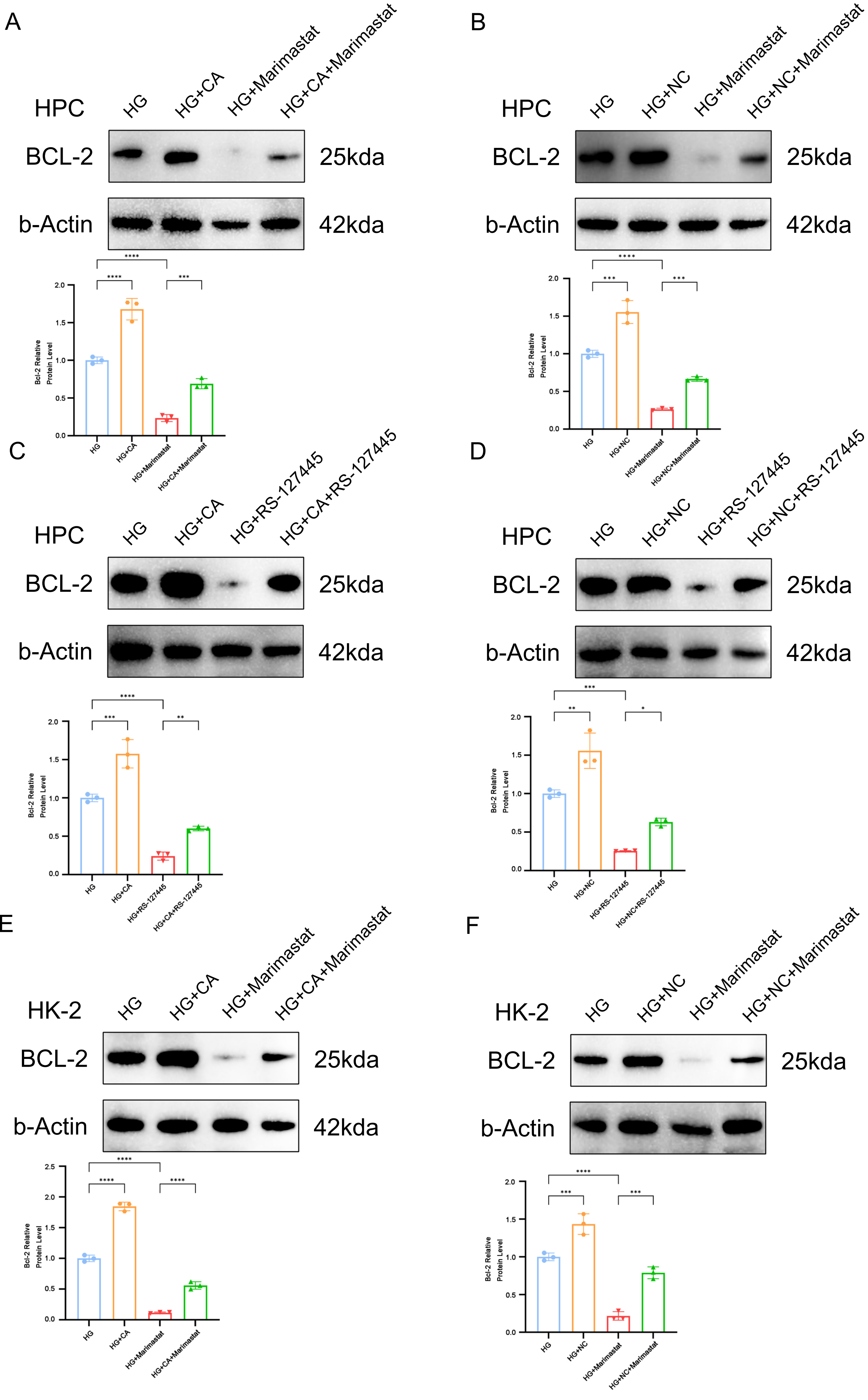
A
HPC
HG
HG+CA
HG+Marimastat
HG+CA+Marimastat
BCL-2
25kda
b-Actin
42kda
Bcl-2 Relative Protein Level
2.0
1.5
1.0
0.5
0.0
****
****
***
HG
HG+CA
HG+Marimastat
HG+CA+Marimastat
B
HPC
HG
HG+NC
HG+Marimastat
HG+NC+Marimastat
BCL-2
25kda
b-Actin
42kda
Bcl-2 Relative Protein Level
2.0
1.5
1.0
0.5
0.0
****
***
***
HG
HG+NC
HG+Marimastat
HG+NC+Marimastat
C
HPC
HG
HG+CA
HG+RS-127445
HG+CA+RS-127445
BCL-2
25kda
b-Actin
42kda
Bcl-2 Relative Protein Level
2.0
1.5
1.0
0.5
0.0
****
***
**
HG
HG+CA
HG+RS-127445
HG+CA+RS-127445
D
HPC
HG
HG+NC
HG+RS-127445
HG+NC+RS-127445
BCL-2
25kda
b-Actin
42kda
Bcl-2 Relative Protein Level
2.0
1.5
1.0
0.5
0.0
***
**
*
HG
HG+NC
HG+RS-127445
HG+NC+RS-127445
E
HK-2
HG
HG+CA
HG+Marimastat
HG+CA+Marimastat
BCL-2
25kda
b-Actin
42kda
Bcl-2 Relative Protein Level
2.5
2.0
1.5
1.0
0.5
0.0
****
****
****
HG
HG+CA
HG+Marimastat
HG+CA+Marimastat
F
HK-2
HG
HG+NC
HG+Marimastat
HG+NC+Marimastat
BCL-2
25kda
b-Actin
Bcl-2 Relative Protein Level
2.0
1.5
1.0
0.5
0.0
****
***
***
HG
HG+NC
HG+Marimastat
HG+NC+Marimastat
