# Supplementary material for: Integrative multi-omics analysis prioritizes compartment-specific candidate targets of gut microbiota metabolites in diabetic kidney disease
Source: Front Immunol. 2026 Aug 31;17:1925910. doi: 10.3389/fimmu.2026.1925910 (PMC13583601; doi:10.3389/fimmu.2026.1925910)
Supplement: Supplementary file 1 [file DataSheet1.zip › Supplementary Figures/figureS2.pdf]

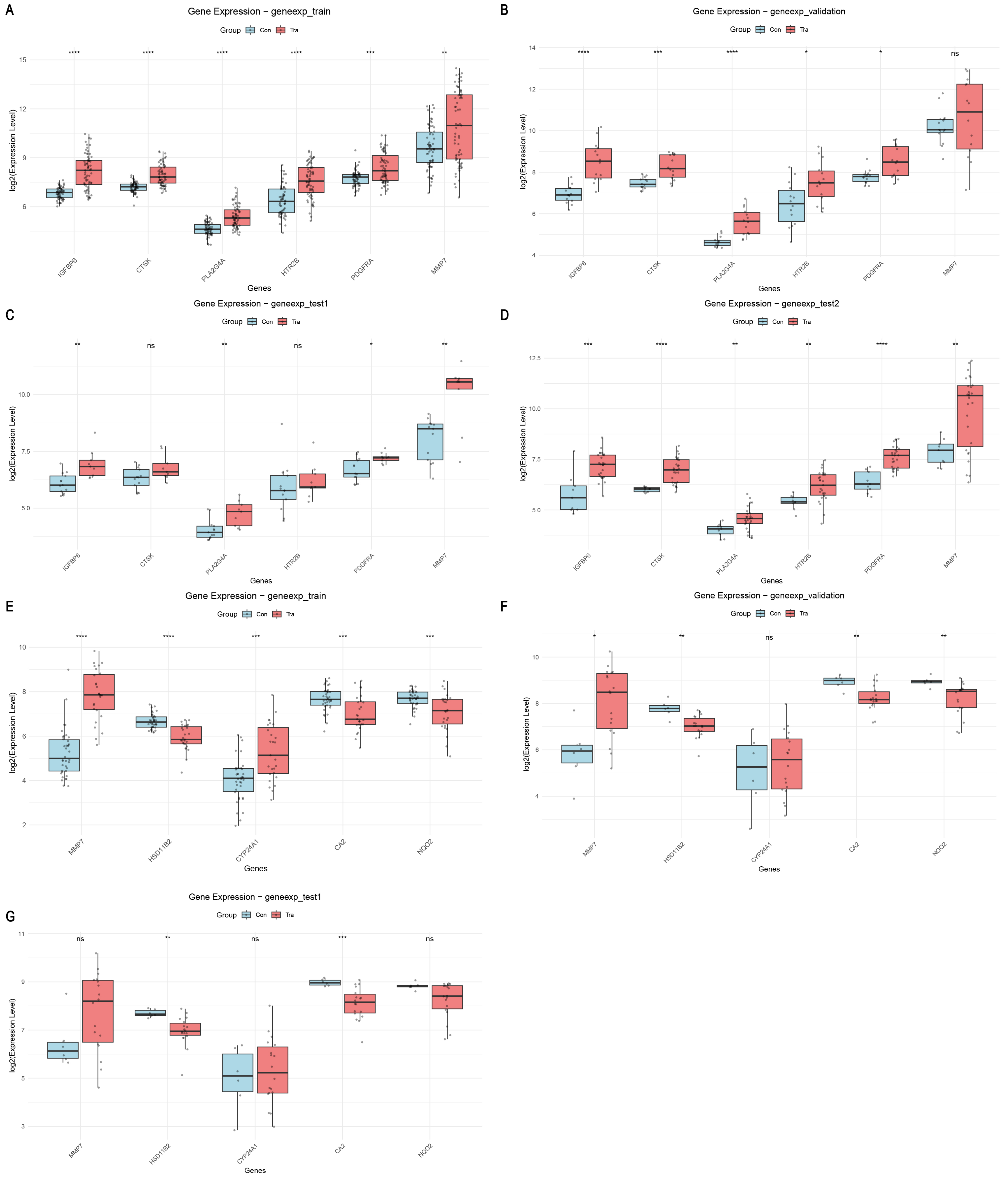
A
Gene Expression − geneexp_train
Group Con Tra
log2(Expression Level)
15
12
9
6
**** **** **** **** *** **
IGFBP6
CTSK
PLA2G4A
HTR2B
PDGFRA
MMP7
Genes
B
Gene Expression − geneexp_validation
Group Con Tra
log2(Expression Level)
14
12
10
8
6
4
**** *** **** * * ns
IGFBP6
CTSK
PLA2G4A
HTR2B
PDGFRA
MMP7
Genes
C
Gene Expression − geneexp_test1
Group Con Tra
log2(Expression Level)
10.0
7.5
5.0
** ns ** ns * **
IGFBP6
CTSK
PLA2G4A
HTR2B
PDGFRA
MMP7
Genes
D
Gene Expression − geneexp_test2
Group Con Tra
log2(Expression Level)
12.5
10.0
7.5
5.0
*** **** ** ** **** **
IGFBP6
CTSK
PLA2G4A
HTR2B
PDGFRA
MMP7
Genes
E
Gene Expression − geneexp_train
Group Con Tra
log2(Expression Level)
10
8
6
4
2
**** **** *** *** ***
MMP7
HSD11B2
CYP24A1
CA2
NQO2
Genes
F
Gene Expression − geneexp_validation
Group Con Tra
log2(Expression Level)
10
8
6
4
* ** ns ** **
MMP7
HSD11B2
CYP24A1
CA2
NQO2
Genes
G
Gene Expression − geneexp_test1
Group Con Tra
log2(Expression Level)
11
9
7
5
3
ns ** ns *** ns
MMP7
HSD11B2
CYP24A1
CA2
NQO2
Genes
