# Supplementary material for: Integrative multi-omics analysis prioritizes compartment-specific candidate targets of gut microbiota metabolites in diabetic kidney disease
Source: Front Immunol. 2026 Aug 31;17:1925910. doi: 10.3389/fimmu.2026.1925910 (PMC13583601; doi:10.3389/fimmu.2026.1925910)
Supplement: Supplementary file 1 [file DataSheet1.zip › Supplementary Figures/figureS3.pdf]

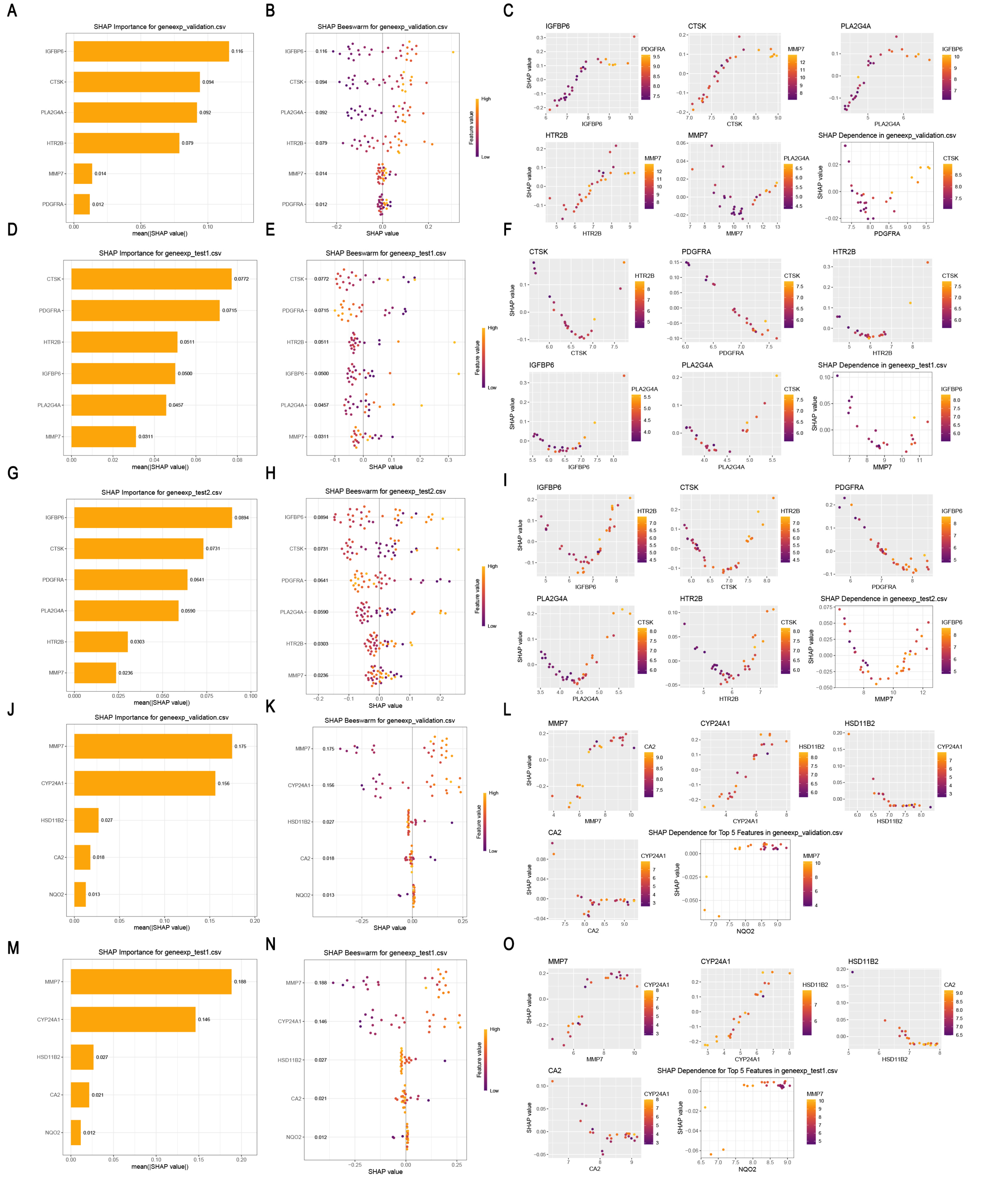
A
SHAP Importance for geneexp_validation.csv
B
SHAP Beeswarm for geneexp_validation.csv
C
SHAP Dependence in geneexp_validation.csv
D
SHAP Importance for geneexp_test1.csv
E
SHAP Beeswarm for geneexp_test1.csv
F
SHAP Dependence in geneexp_test1.csv
G
SHAP Importance for geneexp_test2.csv
H
SHAP Beeswarm for geneexp_test2.csv
I
SHAP Dependence in geneexp_test2.csv
J
SHAP Importance for geneexp_validation.csv
K
SHAP Beeswarm for geneexp_validation.csv
L
SHAP Dependence for Top 5 Features in geneexp_validation.csv
M
SHAP Importance for geneexp_test1.csv
N
SHAP Beeswarm for geneexp_test1.csv
O
SHAP Dependence for Top 5 Features in geneexp_test1.csv
mean(|SHAP value|)
SHAP value
Feature value
High
Low
