# Supplementary material for: Integrative multi-omics analysis prioritizes compartment-specific candidate targets of gut microbiota metabolites in diabetic kidney disease
Source: Front Immunol. 2026 Aug 31;17:1925910. doi: 10.3389/fimmu.2026.1925910 (PMC13583601; doi:10.3389/fimmu.2026.1925910)

A

exposure	outcome	method_label	nsnp	pval_display	OR (95% CI)
CTSK	eGFR (creatinine)	Wald ratio	1	<0.001	0.998 (0.997 to 0.999)
IGFBP6	Chronic kidney disease	Wald ratio	1	0.028	0.700 (0.509 to 0.962)
IGFBP6	Renal failure	Wald ratio	1	0.022	0.739 (0.570 to 0.958)
CYP24A1	Diabetic nephropathy	IVW	2	0.013	0.607 (0.410 to 0.898)
NQO2	eGFR (creatinine)	IVW	3	0.032	0.999 (0.998 to 1.000)

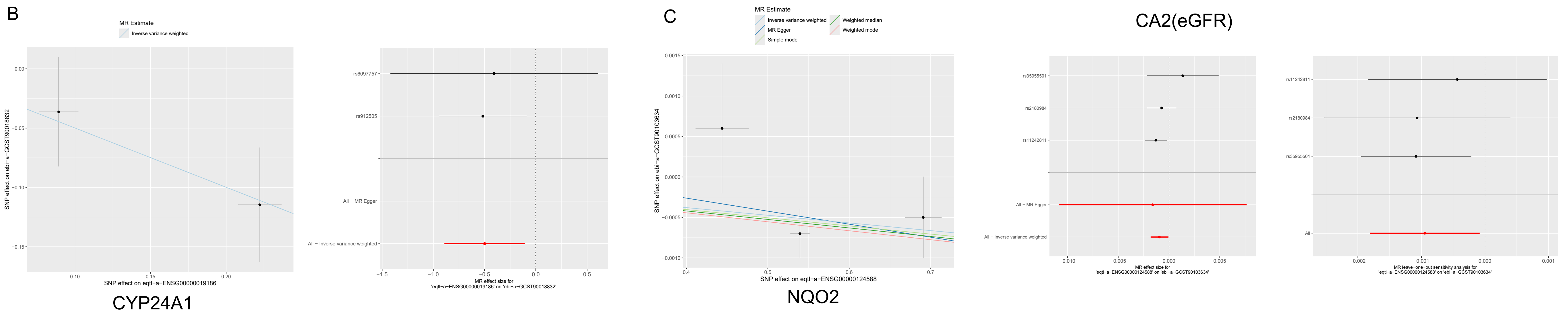

D

exposure	outcome	dataset_display	method_label	nsnp	pval_display	OR (95% CI)
**MMP7**	**eGFR (creatinine)**	**UK Biobank PPP**	**IVW**	**2**	**0.011**	**1.002 (1.001 to 1.004)**
**MMP7**	eGFR (creatinine)	**deCODE**	IVW	**4**	0.0031	1.003 (1.001 to 1.004)
**PDGFRA**	Microalbuminuria	**UK Biobank PPP**	IVW	**2**	0.023	0.823 (0.696 to 0.974)
**PDGFRA**	Renal failure	**UK Biobank PPP**	IVW	**3**	0.046	1.171 (1.003 to 1.368)
**PLA2G4A**	eGFR (creatinine)	**UK Biobank PPP**	**Wald ratio**	**1**	0.046	0.992 (0.985 to 1.000)

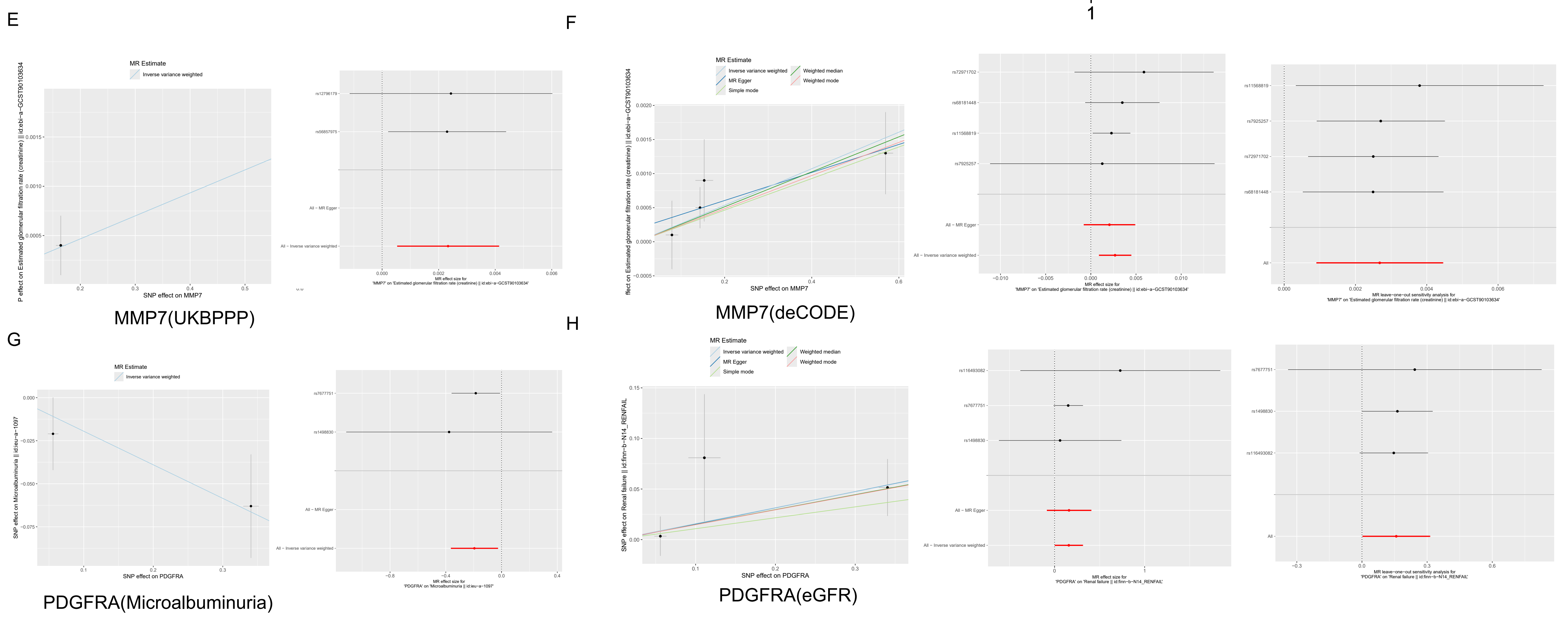

I

exposure	outcome	method_label	nsnp	pval_display	OR (95% CI)
**CTSK**	**Diabetic nephropathy**	**IVW**	**2**	**0.049**	**1.252 (1.001 to 1.566)**
CTSK	eGFR (creatinine)	IVW	2	**0.014**	**0.995 (0.991 to 0.999)**
HSD11B2	eGFR (creatinine)	Wald ratio	1	**<0.001**	**1.038 (1.029 to 1.048)**
HTR2B	Diabetic nephropathy	IVW	2	**0.038**	**1.153 (1.008 to 1.319)**
HTR2B	eGFR (creatinine)	IVW	2	**<0.001**	**0.997 (0.995 to 0.998)**
IGFBP6	Diabetic nephropathy	Wald ratio	1	**0.019**	**0.749 (0.588 to 0.953)**
IGFBP6	eGFR (creatinine)	Wald ratio	1	**0.046**	**0.997 (0.994 to 1.000)**
NQO2	Renal failure	IVW	2	**0.014**	**0.953 (0.918 to 0.990)**
PLA2G4A	eGFR (creatinine)	Wald ratio	1	**<0.001**	**0.987 (0.981 to 0.992)**

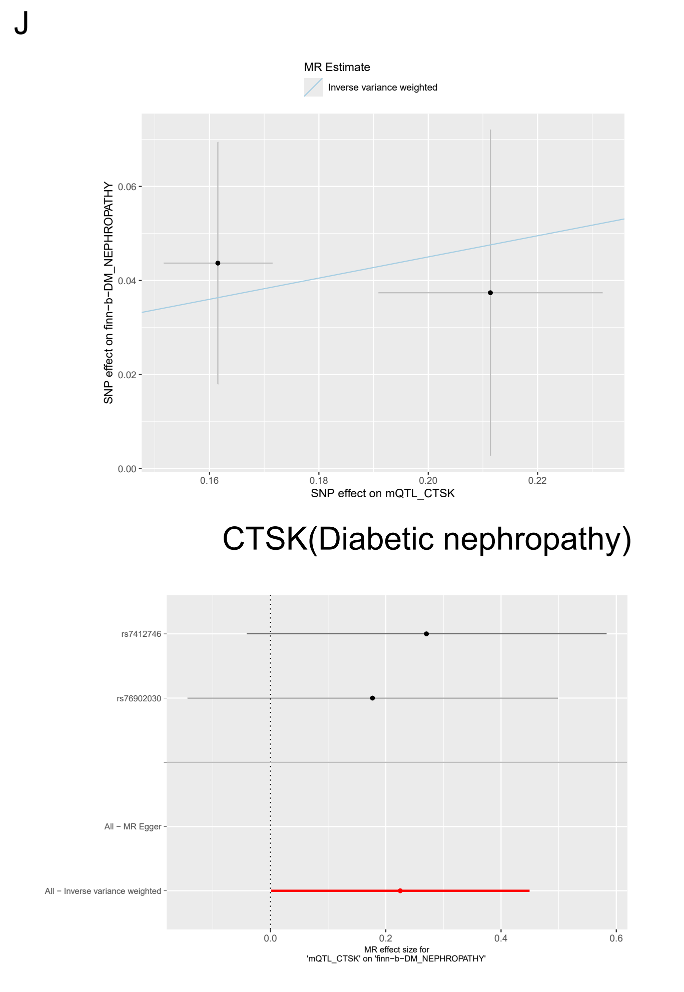

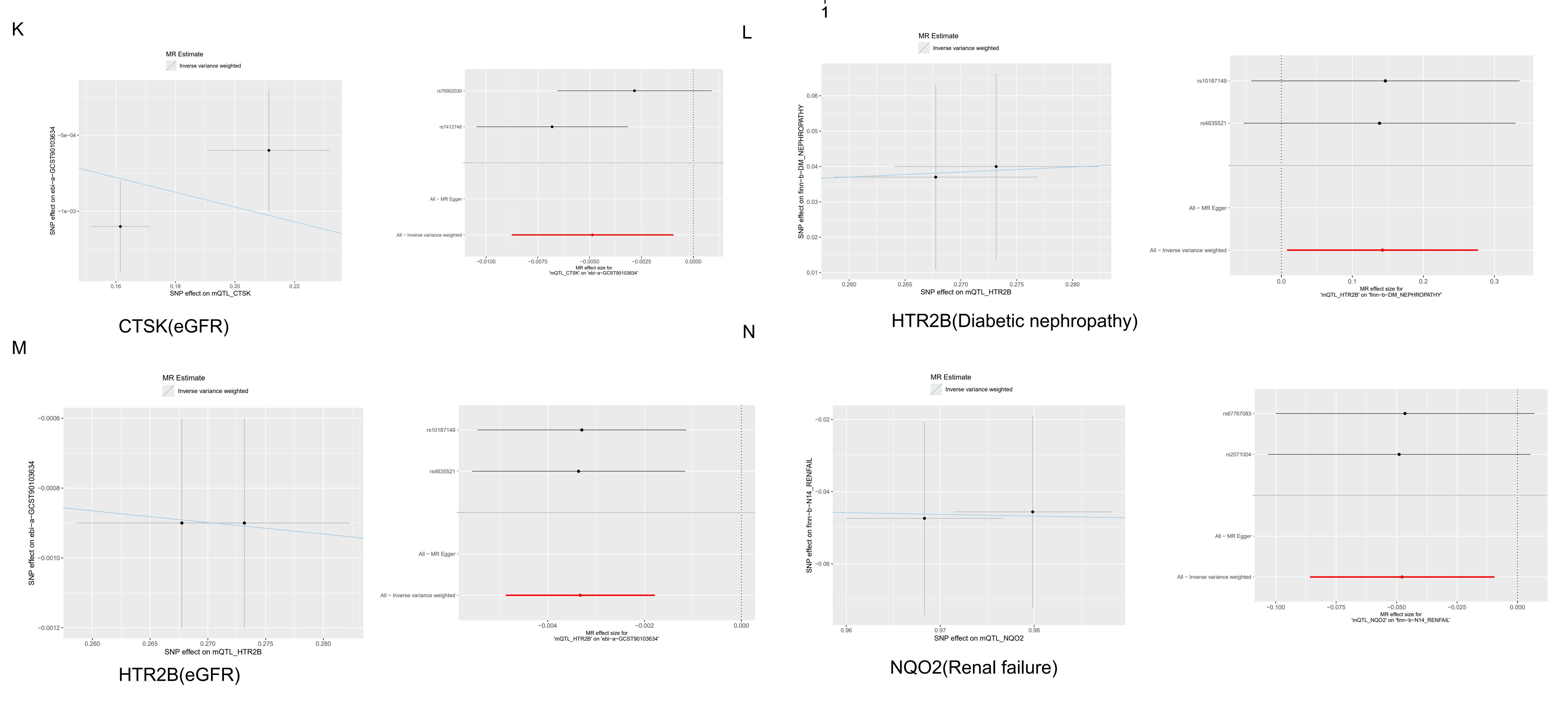

Supplement: Supplementary file 1 [file DataSheet1.zip › Supplementary Figures/figureS4.pdf]
