# Supplementary material for: Integrative multi-omics analysis prioritizes compartment-specific candidate targets of gut microbiota metabolites in diabetic kidney disease
Source: Front Immunol. 2026 Aug 31;17:1925910. doi: 10.3389/fimmu.2026.1925910 (PMC13583601; doi:10.3389/fimmu.2026.1925910)
Supplement: Supplementary file 1 [file DataSheet1.zip › Supplementary Figures/figureS5.pdf]

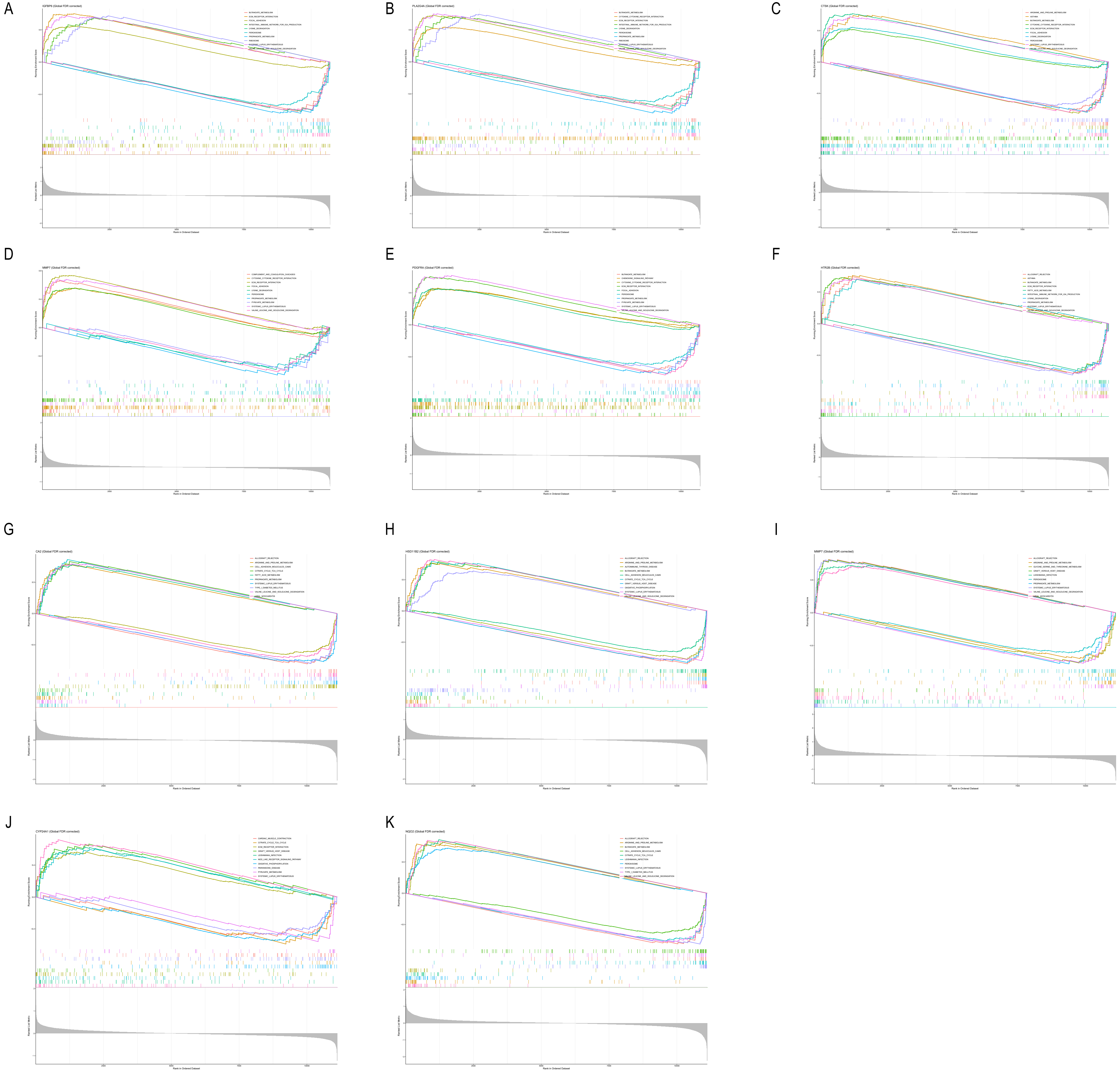
A
IGFBP6 (Global FDR corrected)
B
PLA2G4A (Global FDR corrected)
C
CTSK (Global FDR corrected)
D
MMP7 (Global FDR corrected)
E
PDGFRA (Global FDR corrected)
F
HTR2B (Global FDR corrected)
G
CA2 (Global FDR corrected)
H
HSD11B2 (Global FDR corrected)
I
MMP7 (Global FDR corrected)
J
CYP24A1 (Global FDR corrected)
K
NQO2 (Global FDR corrected)
Running Enrichment Score
Ranked List Metric
Rank in Ordered Dataset
