# Supplementary material for: Integrative multi-omics analysis prioritizes compartment-specific candidate targets of gut microbiota metabolites in diabetic kidney disease
Source: Front Immunol. 2026 Aug 31;17:1925910. doi: 10.3389/fimmu.2026.1925910 (PMC13583601; doi:10.3389/fimmu.2026.1925910)
Supplement: Supplementary file 1 [file DataSheet1.zip › Supplementary Figures/figureS6.pdf]

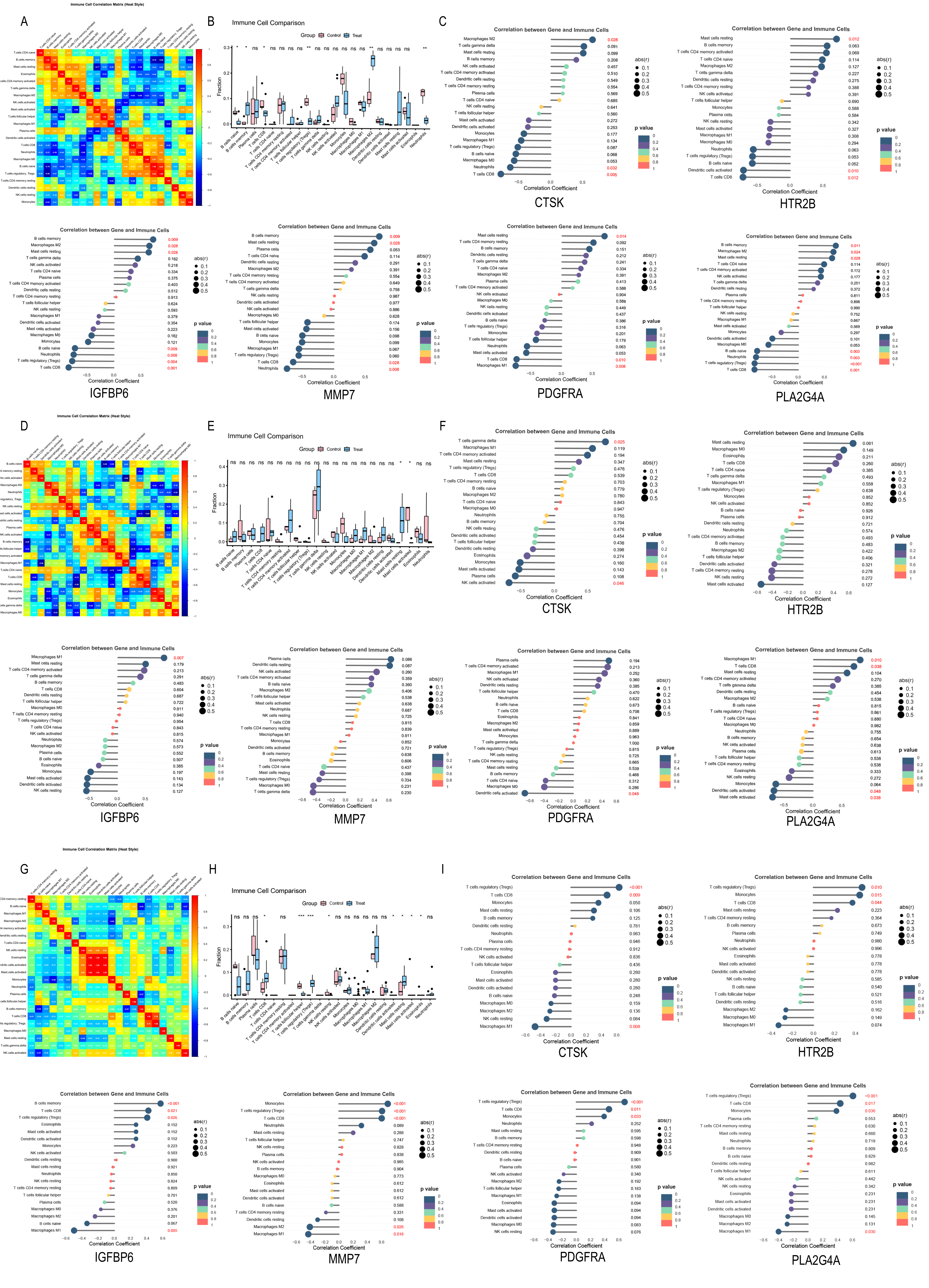
A
Immune Cell Correlation Matrix (Heat Style)
B
Immune Cell Comparison
Group Control Treat
Fraction
C
Correlation between Gene and Immune Cells
Correlation Coefficient
abs(r)
p value
CTSK
HTR2B
IGFBP6
MMP7
PDGFRA
PLA2G4A
D
Immune Cell Correlation Matrix (Heat Style)
E
Immune Cell Comparison
F
CTSK
HTR2B
IGFBP6
MMP7
PDGFRA
PLA2G4A
G
Immune Cell Correlation Matrix (Heat Style)
H
Immune Cell Comparison
I
CTSK
HTR2B
IGFBP6
MMP7
PDGFRA
PLA2G4A
