# Supplementary material for: Integrative multi-omics analysis prioritizes compartment-specific candidate targets of gut microbiota metabolites in diabetic kidney disease
Source: Front Immunol. 2026 Aug 31;17:1925910. doi: 10.3389/fimmu.2026.1925910 (PMC13583601; doi:10.3389/fimmu.2026.1925910)
Supplement: Supplementary file 1 [file DataSheet1.zip › Supplementary Figures/figureS7.pdf]

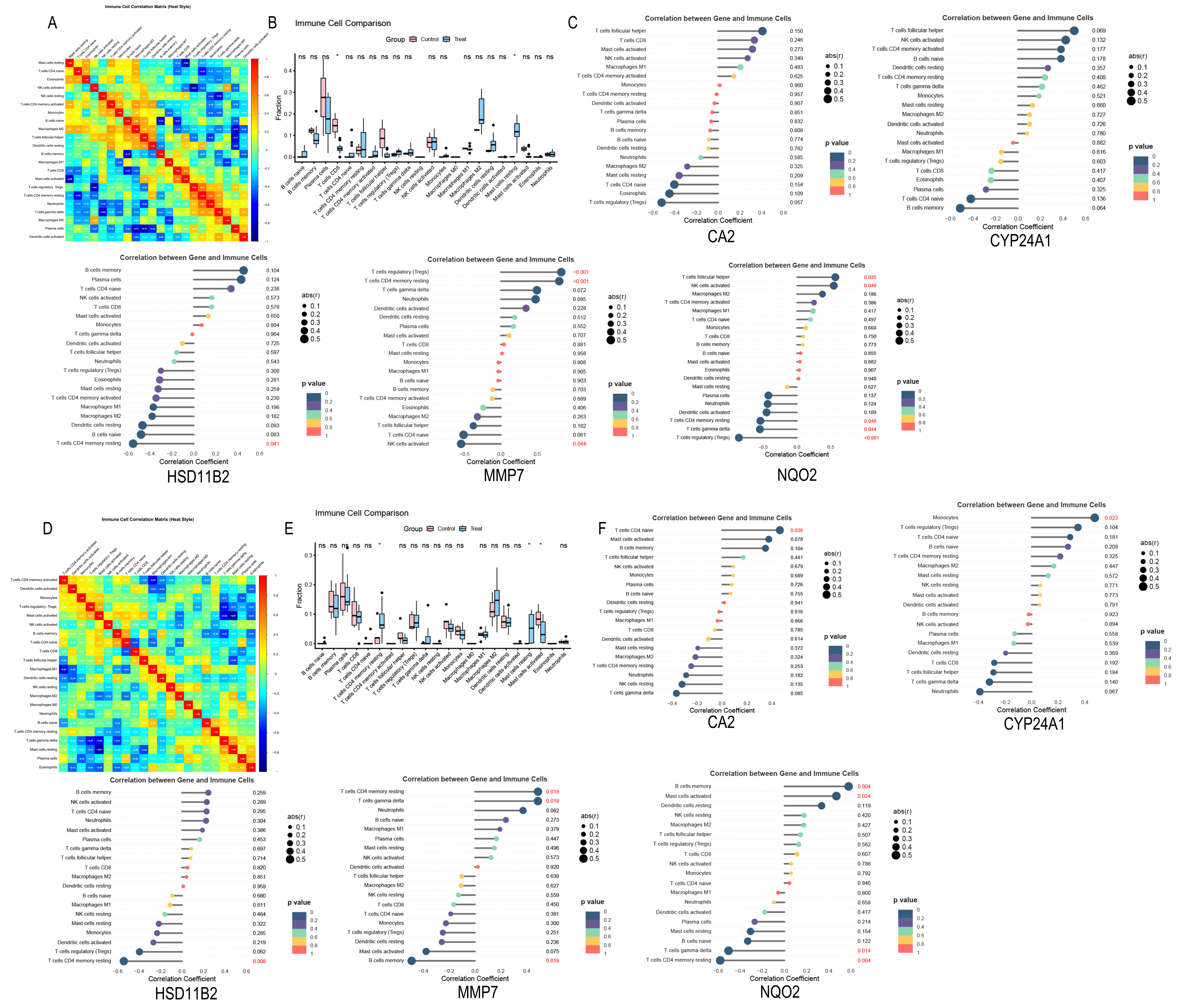
A
Immune Cell Correlation Matrix (Heat Style)
B
Immune Cell Comparison
Group
Control
Treat
Fraction
C
Correlation between Gene and Immune Cells
Correlation Coefficient
abs(r)
p value
CA2
CYP24A1
HSD11B2
MMP7
NQO2
D
Immune Cell Correlation Matrix (Heat Style)
E
Immune Cell Comparison
Group
Control
Treat
Fraction
F
Correlation between Gene and Immune Cells
Correlation Coefficient
abs(r)
p value
CA2
CYP24A1
HSD11B2
MMP7
NQO2
