# Supplementary material for: Integrative multi-omics analysis prioritizes compartment-specific candidate targets of gut microbiota metabolites in diabetic kidney disease
Source: Front Immunol. 2026 Aug 31;17:1925910. doi: 10.3389/fimmu.2026.1925910 (PMC13583601; doi:10.3389/fimmu.2026.1925910)
Supplement: Supplementary file 1 [file DataSheet1.zip › Supplementary Figures/figureS8.pdf]

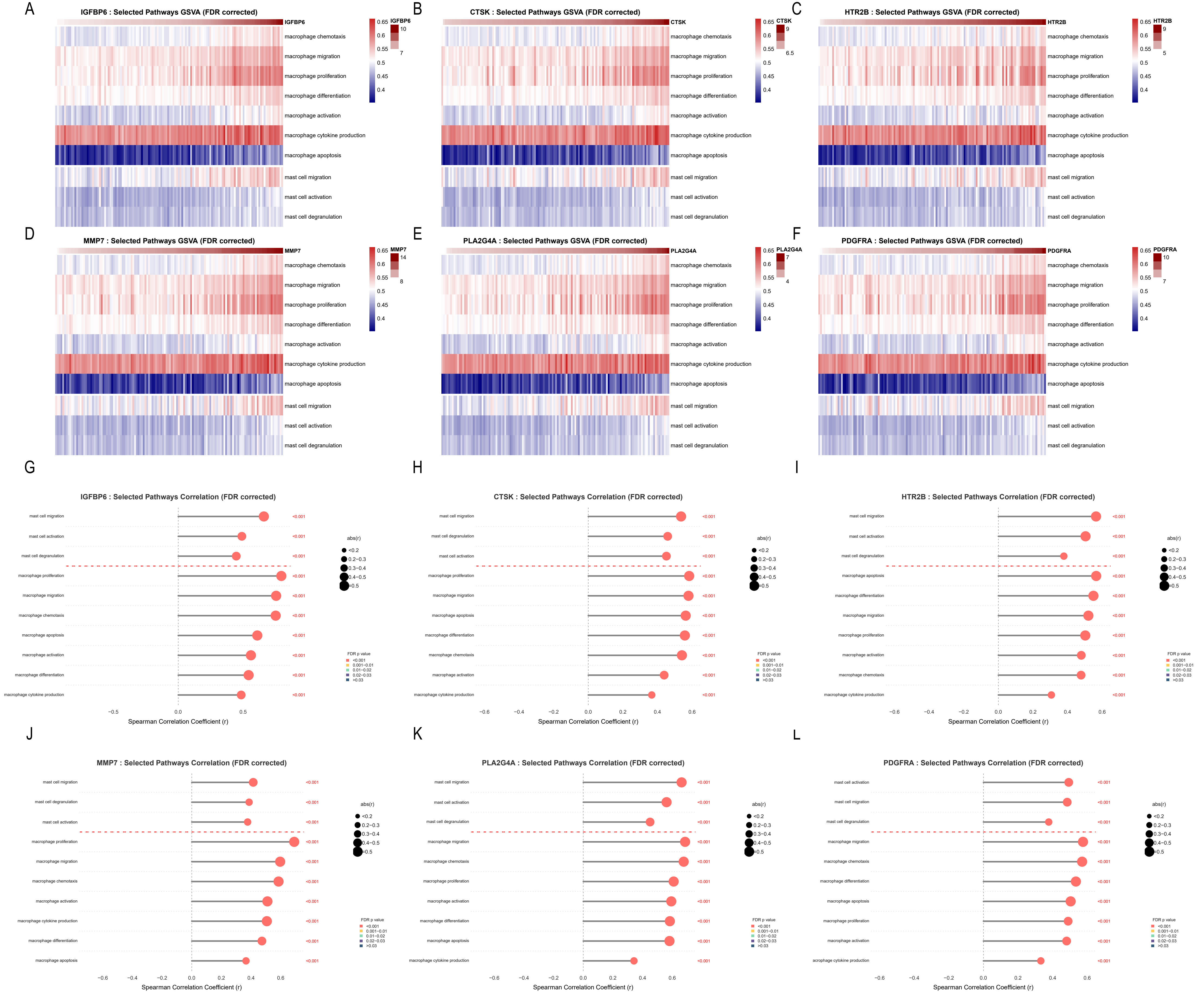

A
IGFBP6 : Selected Pathways GSVA (FDR corrected)
B
CTSK : Selected Pathways GSVA (FDR corrected)
C
HTR2B : Selected Pathways GSVA (FDR corrected)
macrophage chemotaxis
macrophage migration
macrophage proliferation
macrophage differentiation
macrophage activation
macrophage cytokine production
macrophage apoptosis
mast cell migration
mast cell activation
mast cell degranulation
D
MMP7 : Selected Pathways GSVA (FDR corrected)
E
PLA2G4A : Selected Pathways GSVA (FDR corrected)
F
PDGFRA : Selected Pathways GSVA (FDR corrected)
G
IGFBP6 : Selected Pathways Correlation (FDR corrected)
H
CTSK : Selected Pathways Correlation (FDR corrected)
I
HTR2B : Selected Pathways Correlation (FDR corrected)
J
MMP7 : Selected Pathways Correlation (FDR corrected)
K
PLA2G4A : Selected Pathways Correlation (FDR corrected)
L
PDGFRA : Selected Pathways Correlation (FDR corrected)
Spearman Correlation Coefficient (r)
abs(r)
<0.2
0.2~0.3
0.3~0.4
0.4~0.5
>0.5
FDR p value
<0.001
0.001~0.01
0.01~0.02
0.02~0.03
>0.03
