# Supplementary material for: Integrative multi-omics analysis prioritizes compartment-specific candidate targets of gut microbiota metabolites in diabetic kidney disease
Source: Front Immunol. 2026 Aug 31;17:1925910. doi: 10.3389/fimmu.2026.1925910 (PMC13583601; doi:10.3389/fimmu.2026.1925910)
Supplement: Supplementary file 1 [file DataSheet1.zip › Supplementary Figures/figureS9.pdf]

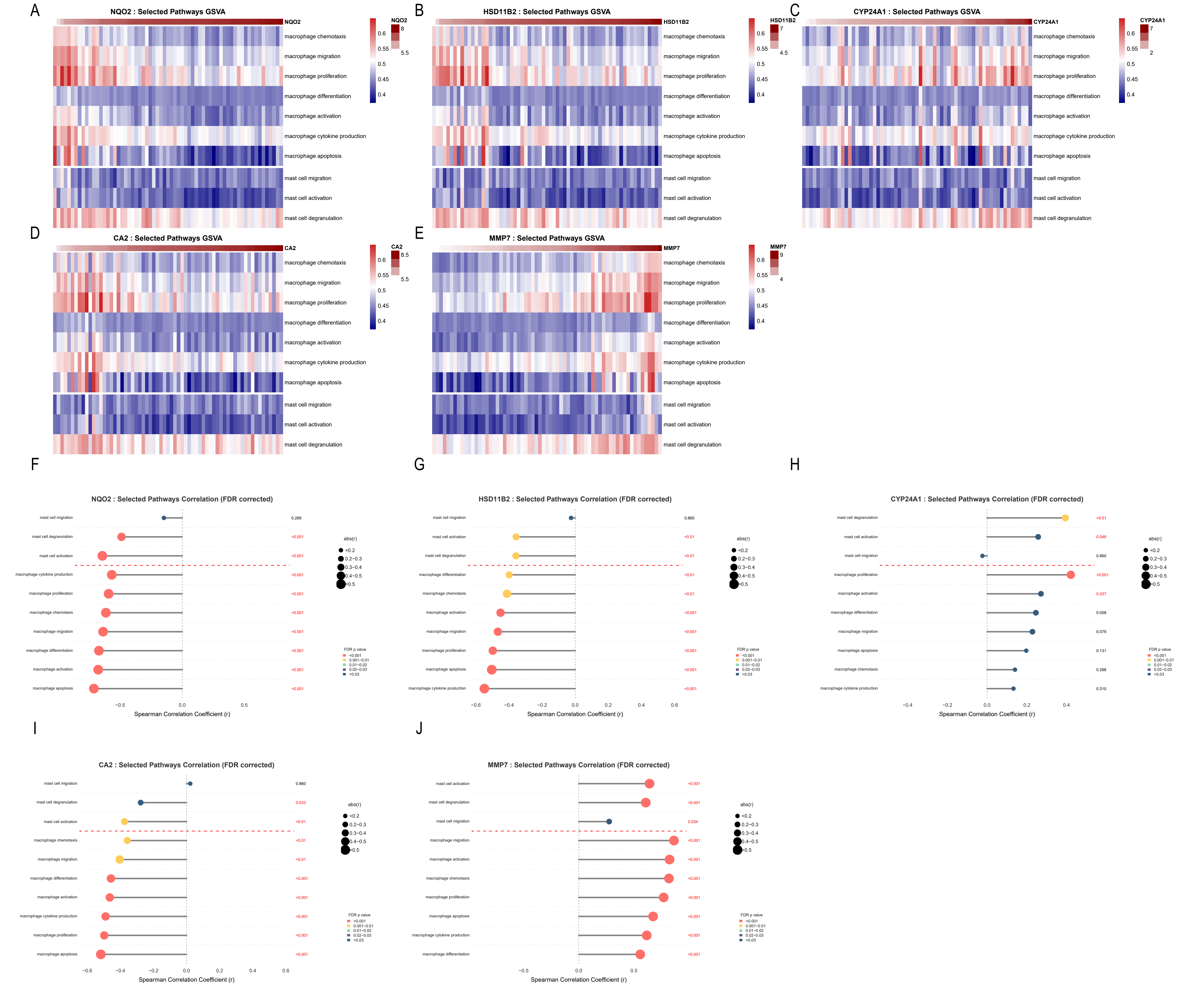
A
NQO2 : Selected Pathways GSVA
B
HSD11B2 : Selected Pathways GSVA
C
CYP24A1 : Selected Pathways GSVA
D
CA2 : Selected Pathways GSVA
E
MMP7 : Selected Pathways GSVA
macrophage chemotaxis
macrophage migration
macrophage proliferation
macrophage differentiation
macrophage activation
macrophage cytokine production
macrophage apoptosis
mast cell migration
mast cell activation
mast cell degranulation
F
NQO2 : Selected Pathways Correlation (FDR corrected)
G
HSD11B2 : Selected Pathways Correlation (FDR corrected)
H
CYP24A1 : Selected Pathways Correlation (FDR corrected)
I
CA2 : Selected Pathways Correlation (FDR corrected)
J
MMP7 : Selected Pathways Correlation (FDR corrected)
abs(r)
<0.2
0.2–0.3
0.3–0.4
0.4–0.5
>0.5
FDR p value
<0.001
0.001–0.01
0.01–0.02
0.02–0.03
>0.03
Spearman Correlation Coefficient (r)
