# Supplementary material for: Integrative multi-omics analysis prioritizes compartment-specific candidate targets of gut microbiota metabolites in diabetic kidney disease
Source: Front Immunol. 2026 Aug 31;17:1925910. doi: 10.3389/fimmu.2026.1925910 (PMC13583601; doi:10.3389/fimmu.2026.1925910)
Supplement: Supplementary file 1 [file DataSheet1.zip › Supplementary Figures/Supplementary Figure Legends.docx]

Figure S1. WGCNA Analysis of the Training Sets.

(A) Selection of the soft-thresholding power (β) for the glomerular and (C) tubulointerstitial cohorts. The plot displays the changes in the scale-free topology fit index (R^2^) and mean connectivity under various β values.

(B) Gene dendrogram generated by hierarchical clustering, with colors corresponding to the assigned gene modules, for the glomerular and (D) tubulointerstitial cohorts.

Figure S2. Box plots of core gene expression across different datasets.

(A-D) Box plots showing the expression levels of the six core genes (*IGFBP6*, *PLA2G4A*, *CTSK*, *HTR2B*, *PDGFRA*, and *MMP7*) in the glomerular datasets.

(E-G) Box plots showing the expression levels of the five core genes (*CA2*, *HSD11B2*, *NQO2*, *MMP7*, and *CYP24A1*) in the tubulointerstitial datasets.

The x-axis displays the gene names, and the y-axis represents the log2(Expression Level). Statistical significance: ns: p-value ≥ 0.05.*: p-value < 0.05.**: The p-value <0.01.***: The p-value <0.001.****: The p-value <0.0001.

Figure S3. SHAP analysis in the validation and test sets. (A) SHAP summary plots for the glomerular validation set, (D, G) glomerular test set, (J) tubulointerstitial validation set, and (M) tubulointerstitial test set. (B) Beeswarm plots for the glomerular validation set, (E, H) glomerular test set, (K) tubulointerstitial validation set, and (N) tubulointerstitial test set. (C) SHAP dependence plots for the glomerular validation set, (F, I) glomerular test set, (L) tubulointerstitial validation set, and (O) tubulointerstitial test set.

Figure S4. Mendelian randomization analysis of core genes on DKD-related traits.
(A) Forest plot of nominally significant eQTL associations with DKD-related traits.
(B–E) Sensitivity analyses for eQTLs: Scatter and forest plots are shown for CA2 on dialysis (B) and eGFR (C) and CYP24A1 on diabetic nephropathy (D); panel (E) additionally includes leave-one-out analysis for NQO2 on eGFR.
(F) Forest plot of nominally significant pQTL associations with DKD-related traits.
(G–J) Sensitivity analyses for pQTLs: Scatter and forest plots are shown for MMP7 (UKBPPP) on eGFR (G) and PDGFRA on microalbuminuria (I) and renal failure (J); panel (H, J) additionally includes leave-one-out analysis for MMP7 (deCODE) on eGFR.

Associations are considered nominally significant at P < 0.05.

Figure S5. Single-gene GSEA of all core genes in the glomerular and tubulointerstitial training sets, showing the top 5 positively and negatively enriched KEGG pathways for each gene.

Figure S6. Immune cell infiltration analysis of core genes in the glomerular validation and test sets.

(A) Correlation analysis of 22 immune cell infiltration levels in the validation set and (D, G) test sets. (B) Box plot comparing the infiltration fractions of 22 key immune cells in the validation set and (E, H) test sets. (C) Lollipop plots illustrating the relationship between core genes and immune cells in the validation set and (F, I) test sets. Red numbers indicate significant correlations, while black numbers indicate non-significant correlations.

Figure S7. Immune cell infiltration analysis of core genes in the tubulointerstitial validation and test sets.

(A) Correlation analysis of 22 immune cell infiltration levels in the validation set and (D) test set. (B) Box plot comparing the infiltration fractions of 22 key immune cells in the validation set and (E) test set. (C) Lollipop plots illustrating the relationship between core genes and immune cells in the validation set and (F) test set. Red numbers indicate significant correlations, while black numbers indicate non-significant correlations.

Figure S8. GSVA of glomerular core genes in the glomerular training set. (A–F) Heatmaps of GSVA enrichment scores for macrophage- and mast cell‑related pathways (adjusted P < 0.005). (G–L) Lollipop plots showing correlations between core gene expression and GSVA enrichment scores; red indicates significant correlations (adjusted P < 0.005), black indicates non‑significant.

Figure S9. GSVA of tubulointerstitial core genes in the tubulointerstitial training set. (A–E) Heatmaps of GSVA enrichment scores for macrophage- and mast cell‑related pathways (adjusted P < 0.005). (F–J) Lollipop plots showing correlations between core gene expression and GSVA enrichment scores; red indicates significant correlations (adjusted P < 0.005), black indicates non‑significant.

Figure S10. Single-cell Analysis of Core Genes in DKD.

(A-D) Single-cell expression patterns of core genes (IGFBP6, PLA2G4A, CTSK, HTR2B, and MMP7) in the DKD and control glomeruli. (F-J) Single-cell expression patterns of core genes (IGFBP6, PLA2G4A, CTSK, HTR2B, and MMP7) in the DKD and control renal tubulointerstitium. Violin plots illustrate the gene expression levels, while bubble plots show the percentage of cells expressing the gene in each cell type. These results were generated based on the GSE131882 single-cell dataset analysis. Cell type abbreviations (GSE131882): PODO, podocytes; MES, mesangial cells; PEC, parietal epithelial cells; PCT, proximal convoluted tubule; LOH, loop of Henle; DCT, distal convoluted tubule; CT, connecting tubule; CD, collecting duct; PC, principal cells; IC, intercalated cells; ENDO, endothelial cells; LEUK, leukocyte.

(E) Single-cell expression pattern of PDGFRA. The size of the bubble represents the percentage of cells expressing the gene, and the color gradient represents the average expression level. This result was generated based on the GSE195460 single-nucleus RNA sequencing (snRNA-seq) dataset analysis. Cell type abbreviations (GSE195460): PT, proximal tubule; PTVCAM1, VCAM1(+) proximal tubule; PEC, parietal epithelial cells; ATL, ascending thin limb; TAL1, CLDN16(-) thick ascending limb; TAL2, CLDN16(+) thick ascending limb; DCT1, early distal convoluted tubule; DCT2, late distal convoluted tubule; PC, principal cell; ICA, type A intercalated cells; ICB, type B intercalated cells; PODO, podocytes; ENDO, endothelium; MES, mesangial cells; FIB, fibroblasts; LEUK, leukocytes.

Figure S11. Correlation Analysis of Core Genes with Renal Function in DKD.

(A-F) Scatter plots showing the relative expression levels of core genes versus the estimated Glomerular Filtration Rate (eGFR), derived from the “Woroniecka Diabetes Glom” dataset in the Nephroseq 5.0 database (corresponding to the glomerular cohort).

(G-K) Scatter plots showing the relative expression levels of core genes versus eGFR, derived from the “Woroniecka Diabetes Tublnt” dataset in the Nephroseq 5.0 database (corresponding to the tubulointerstitial cohort).

All gene expression levels were median-centered log2 transformed. Scatter plots display the fitted linear regression model. Red dots represent DKD patients, and grey dots represent the control group. The solid green line is the fitted linear regression curve, and the light green area represents the 95% confidence interval. The annotated R value is the Pearson correlation coefficient, and the P-value tests for statistical significance.

Figure S12. The 2D structure of naringenin chalcone and caffeic acid.

Figure S13. Identification of structures for naringenin chalcone and caffeic acid.

(A) Chemical structure of caffeic acid. (B) Chemical structure of naringenin chalcone.

Figure S10. Molecular Dynamics Simulation of the MMP7-Naringenin Chalcone, HTR2B-Naringenin Chalcone, and HTR2B-Caffeic Acid Complexes.

(A-C) Dominant residue interaction maps. (D-F) Time-dependent curves showing the number of hydrogen bonds for each of the three complexes. (G-I) Three-Dimensional Free Energy Landscapes (3D FELs) of the three complexes. (J-L) Two-Dimensional Free Energy Landscapes (2D FELs) of the three complexes.

Figure S14. Western blot analysis of MMP7, HTR2B (in podocytes only), and Bcl‑2 protein expression in HK‑2 cells and podocytes under normal glucose, mannitol, high glucose (HG), and HG co‑treated with caffeic acid or naringenin chalcone. β‑actin was used as the loading control. Bar graphs show densitometric quantification of protein expression levels normalized to β‑actin. Data are presented as mean ± SD (n = 3). *P < 0.05, **P < 0.01, **P < 0.001.

Figure S15. Western blot analysis of Bcl‑2 expression in HK‑2 cells and podocytes under normal glucose, HG, HG with pharmacological modulation of MMP7 (inhibition) or HTR2B (inhibition), and HG with target modulation plus caffeic acid or naringenin chalcone. β‑actin was used as the loading control. Bar graphs show densitometric quantification of Bcl‑2 expression normalized to β‑actin. Data are presented as mean ± SD (n = 3). *P < 0.05, **P < 0.01, **P < 0.001.
